# Aspartic protease inhibitor enhances resistance to potato virus Y and A in transgenic potato plants

**DOI:** 10.1186/s12870-022-03596-8

**Published:** 2022-05-12

**Authors:** Zhila Osmani, Mohammad Sadegh Sabet, Kenji S. Nakahara

**Affiliations:** 1 Department of Plant Genetics and Breeding, Faculty of Agriculture, Tarbiat Modares University, Tehran, 14111713116 Iran; 2 Research Faculty of Agriculture, Hokkaido University, Sapporo, Japan

**Keywords:** Protease inhibitor, Virus resistance, Defense response, Overexpression, Potato

## Abstract

**Background:**

Viruses are the major threat to commercial potato (*Solanum tuberosum*) production worldwide. Because viral genomes only encode a small number of proteins, all stages of viral infection rely on interactions between viral proteins and host factors. Previously, we presented a list of the most important candidate genes involved in potato plants’ defense response to viruses that are significantly activated in resistant cultivars. Isolated from this list, Aspartic Protease Inhibitor 5 (*API5*) is a critical host regulatory component of plant defense responses against pathogens. The purpose of this study is to determine the role of *StAPI5* in defense of potato against potato virus Y and potato virus A, as well as its ability to confer virus resistance in a transgenic susceptible cultivar of potato (Desiree). Potato plants were transformed with *Agrobacterium tumefaciens* via a construct encoding the potato *StAPI5* gene under the control of the Cauliflower mosaic virus (CaMV) 35S promoter.

**Results:**

Transgenic plants overexpressing *StAPI5* exhibited comparable virus resistance to non-transgenic control plants, indicating that *StAPI5* functions in gene regulation during virus resistance. The endogenous *StAPI5* and CaMV 35S promoter regions shared nine transcription factor binding sites. Additionally, the net photosynthetic rate, stomatal conductivity, and maximum photochemical efficiency of photosystem II were significantly higher in virus-infected transgenic plants than in wild-type plants.

**Conclusion:**

Overall, these findings indicate that *StAPI5* may be a viable candidate gene for engineering plant disease resistance to viruses that inhibit disease development.

**Supplementary Information:**

The online version contains supplementary material available at 10.1186/s12870-022-03596-8.

## Background

Modern agriculture must ensure a sufficient supply of nutritious food for the world’s growing population, which is expected to reach nearly 10 billion by 2050, 35% increase over today’s level. In their natural environment, plants are constantly exposed to various biotic and abiotic stresses, which affect their growth, development, and productivity. Potato (*Solanum tuberosum* L.) is the world’s most widely grown non-grain staple crop and the fourth most important food crop after maize, rice, and wheat (FAOSTAT, www.faostat.fao.org). Previous research has shown that viruses can alter leaf morphology, reduce plant vegetative performance, cause a dramatic decrease in photosynthesis, and increase respiration. These adverse effects of viral infection on plant development result in lower crop quality and yield loss [1, 2]. Viruses, particularly potyviruses like Potato Virus Y and A (PVY and PVA), cause significant economic losses in potato seed production today, making it a critical major pathogen threatening global food security [3]. PVY and PVA infection rates have been observed to range between 50 and 100% depending on the viral strain, the time of infection, the stage of the plant, and the rate of spread.

The study and understanding of the molecular mechanisms underlying plants’ interactions with pathogens are necessary to develop sources of resistance in the face of global environmental change. It enables us to understand better how pathogens attack plants’ immune defenses. Successful interaction between plants and viruses is dependent on complex molecular mechanisms, including the viral hijacking of essential plant factors for infection, plant defense antiviral mechanisms that inhibit viral infections, and viral counter defense mechanisms that circumvent plant defense [4, 5]. Depending on the potato cultivars, environmental factors, viral strains, and time of infection, the virus can cause different infection levels with varying outcomes [5].

Recessive resistance can occur due to changes in the host factors required for the virus [4, 6]. Plant viruses must replicate and translocate through plasmodesmata between cells to spread systemically throughout the plant’s vascular system [7, 8]. Due to the small size of RNA virus genomes, they only encode a small number of proteins required for viral infection. Thus, the virus must efficiently exploit host components to initiate the infection cycle [8, 9]. Manipulation of plant factors required for the virus life cycle has been shown to be an effective method of combating plant viruses in various crop species, but only a few have been identified [9, 10]. Although R-gene-mediated resistance generally results in localized programmed cell death, termed the hypersensitive response (HR), this resistance perceives specific pathogen effectors (race-specific resistance) [11].

As a result, R-gene-mediated resistance frequently deteriorates in the field after a few years of use due to virulence changes in the pathogen population [12]. Thus, for pathogen resistance breeding, the emphasis is on using genes involved in pathogenesis pathways and innate immune responses in plants via transgenic-based approaches such as overexpression tests [13]. Unlike breeding through the introduction of R genes, genetic manipulation of pathogenesis-related genes such as signal transduction and downstream defense response genes can promote effective and long-lasting defense in plants [13–16], including protease inhibitors (PIs).

The findings indicate that the PIs, significant class of PR proteins, are involved in inhibiting pathogen-secreted proteolytic enzymes (toxic or anti-metabolic compounds) [17]. There are four major classes of PIs based on the active site of the target protease: Ser protease inhibitors, Cys protease inhibitors, aspartic protease inhibitors, and metallo-protease inhibitors [18]. Several PIs have been isolated and transferred from diverse sources into other plant species to generate pathogen-resistant transgenic plants [19–21]. A rice cysteine proteinase inhibitor’s expression in tobacco plants showed significant resistance to two essential potyviruses (Tobacco Etch virus; TEV and PVY) [22].

Aspartic protease inhibitors (*API*s) are proteins that inhibit the catalytic activity of aspartyl proteases, a subclass of proteases defined by the presence of an aspartate residue (Asp) in the active site [18]. *API*s are critical for maintaining normal physiology and life cycles of eukaryotic and prokaryotic systems. Additionally, it is well-established that Aspartic proteases can mediate the initial invasion steps of infectious organisms [23, 24]. Although extensive research has been conducted on the biochemical and kinetic properties of aspartic proteases and their inhibitors in microbial and mammalian systems, limited research has been conducted on plants.

There are numerous reports on serine, cysteine, and metalloprotease inhibitors, but there is a dearth of data on *API*s derived from plants. Viral proteases convert the polyproteins encoded by the potyvirus genome into mature proteins [25]. Furthermore, PIs binding to the active site of HIV-1 protease will be able to inhibit the virus replication cycle [26]. In their viral genomes, retroviruses and HIV-1 protease both encode aspartic proteases [27, 28]. It is hypothesized that host *API*s represent a novel strategy for viral infection control. Due to their critical roles in human diseases (breast cancer metastasis, malaria, and AIDS), far more attention has been paid to aspartic proteases, including cathepsin D, plasmepsins, and HIV-1 peptidase [18]. *API*s are less prevalent and reported in only a few plant species, such as the potato [18]. There is only a single class of aspartic proteases in potato tuber plants. Although this family primarily comprises serine protease inhibitors, it has been demonstrated to inhibit cathepsin, an aspartic protease known as the *API* family [29].

Our previous work identified a set of 265 genes involved in resistance to PVY and PVA in potato plants through meta-analysis of publicly available microarray data sets. In the current study, we have tested whether overexpressing of pathogenesis-related genes, including protease inhibitors can promote effective defense in plants against viruses. We successfully cloned a novel aspartic protease inhibitor (GenBank accession number MH686153, API5-like protein (*StAPI5*)) from potato (*Solanum tuberosum* L. cv. Desiree), which was previously shown to have a significantly more robust response to various virus infections (PVY, PVA, and PLRV) [30]. We generated transgenic potato (cv. Desiree) plants overexpressing *StAPI5* and investigated the abilities of the transgenic plants to resist PVY and PVA infection. PVY and PVA infection. Subsequently, the transgenic plants challenged with PVY and PVA increased resistance to PVY and PVA by retarding disease development in potato plants.

## Results

### Selection of candidate gene

**Fig. 1:**
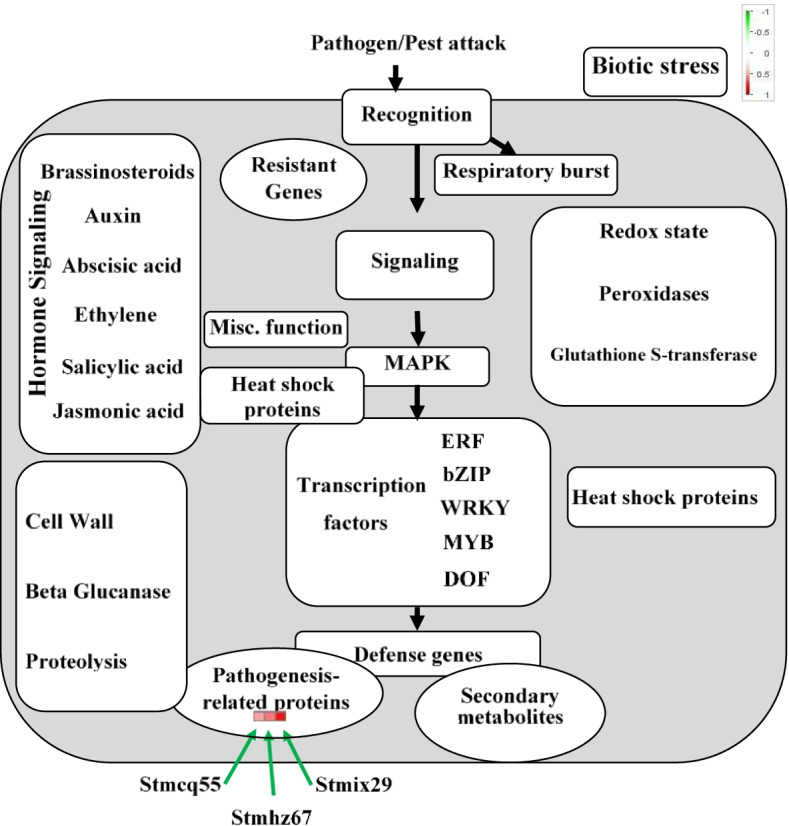
MapMan visualization of the three screened PR-proteins in pathogen/pest attack and biotic stress pathway. Each colored square represents log2 ratio of the expression of one gene (virus infected vs. mock). red, up-regulated; green, down-regulated

Our previous research identified and classified a set of candidate essential genes (265 genes) [30]. The analysis of these 265 genes indicated the presence of eight PR proteins, three of which were proteinase inhibitors (Table 1). The results revealed that eight PR proteins play a role in virus resistance gene regulation. Virus stresses increase transcript levels in all cases (Table 1), and these genes respond via downstream pathways (Fig. 1) [13, 16, 31, 32]. Among the three proteinase inhibitors, the stmix29 and stmhz67 genes have the potential to interfere with other stresses to, thereby increasing resistance to virus stresses (Table 1) (inducing more or less susceptibility to other biotic and abiotic stresses). As demonstrated by the results, the stmcq55 gene expression changes in response to other biotic and abiotic stresses were comparable to virus stresses. Thus, in this study, we chose to overexpress stmcq55, a proteinase inhibitor, in potato plants to confer resistance to virus stresses.

**Table 1:**
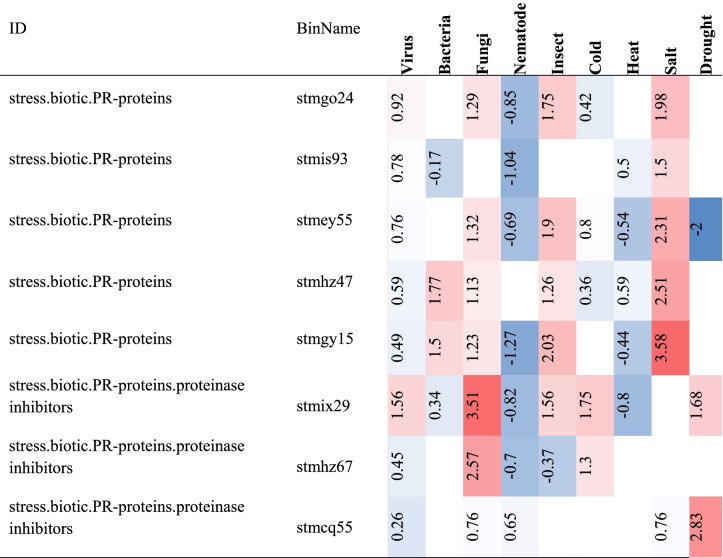
The HeatMap of the 8 pathogenesis-related (PR) proteins screened from microarray meta-analysis in response to different stresses in potato plants. Expression values in each cell are log2-transformed ratios of different stresses/control samples. Red color indicated up- and blue down regulated genes, and white no significant change

### Detection of transgenic events in potato plants

**Fig. 2:**
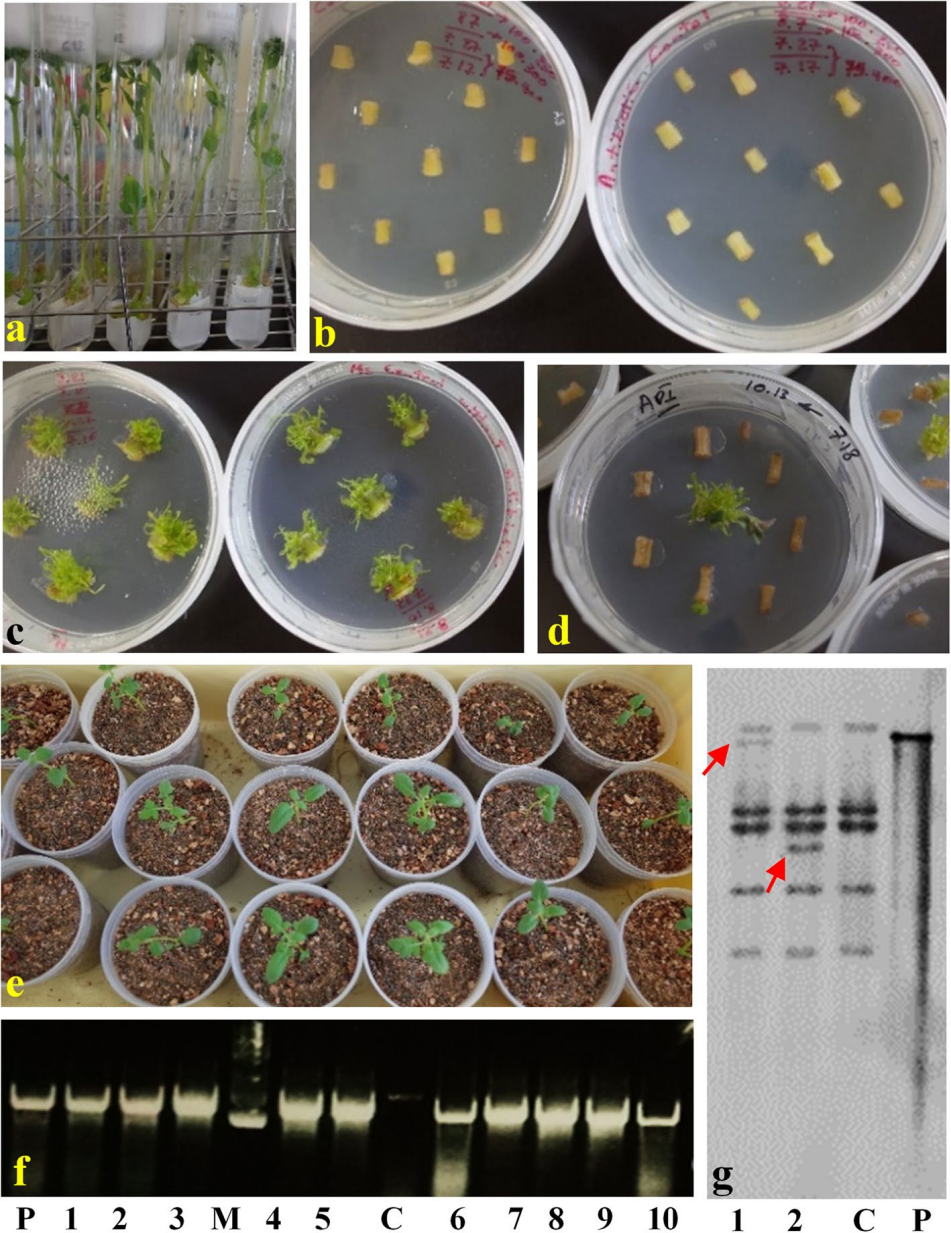
Transformation and molecular analysis of putative transgenic potato plants. **a** In vitro propagation of potato plants from culture of nodal segments on liquid MS medium, **b** No shoot regeneration from the cultured internodes on control selective MS medium containing antibiotics; **c** Complete direct shoot regeneration (100%) from non-inoculated internode pieces on control medium without antibiotic **d** Efficient direct shoot regeneration rate (35%) from inoculated internode segments with Agrobacterium harboring the constructs on the selective medium supplemented with antibiotics, **e** The young plants obtained from independent regeneration events, **f** PCR analysis of DNA isolated from transgenic plants with CaMV 35S forward primer (35S-F) and *StAPI5* gene reverse specific primer (*StAPI5*-R). The expected band length is 754-bp. C: Negative control with non-transgenic potato plant (wild-type); P: positive control PCR reaction with plasmid as template; Lanes no. 1–10: DNA from independent transgenic potato plants and M: 1 kb DNA Ladder. **g** Southern blot analysis of the independent transgenic potato plants overexpressing *StAPI5*-OE gene. P: the pRI-AN201 binary vector containing the *StAPI5* gene (positive control); lanes 1 and 2: independent transgenic potato plants; C: non-transformed potato (negative control); the arrows represent the random integration of transgene(s) into the host genome of transgenic potato plants

Confirmed colonies from the destination vector (Additional file 1: Fig. S1) were used for transformation. The *StAPI5* gene was overexpressed into the susceptible potato cultivar Desiree via agrobacterium-mediated transformation to determine whether it influenced viral resistance. After cultivating single-node cuttings in a liquid propagation medium (Fig. 2a), the internode pieces of the explants were transformed with *StAPI5*-containing constructs. MS control medium containing kanamycin but not cefotaxime inhibited non-transformed internodes regeneration (Fig. 2b). In comparison, all non-transformed internode segment explants were regenerated without antibiotics in control culture media (with 100% regeneration efficiency, Fig. 2c). A selective medium containing kanamycin was used to screen transgenic plants and achieved a transformation efficiency of 35% (Fig. 2d). Forty-three independent regenerants were obtained: 9 with the vector alone (control) and 34 with the *StAPI5*-OE construct (Fig. 2e). Moreover, PCR was used to determine whether T-DNA was inserted into the genomes of transformed potato plants using the CaMV 35 S forward primer (35S-F) and the *StAPI5* gene reverse specific primer (*StAPI5*-R) with product 754 bp (Fig. 2f, Additional file 2: Fig. S2). Genomic PCR analysis revealed that the ten independently regenerated plants possessed the expected bands.

Southern blot analysis was also used to confirm the gene insertion patterns in the screened transgenic plants. The results indicated that the expression cassette was successfully inserted into the potato genome (all data not shown), whereas non-transgenic control plants did not exhibit any transgene. The variable size of the target gene on the blot is due to an independent pattern of transgene insertion into the various genome locations of transgenic potato plants (Fig. 2g, Additional file 3: Fig. S3). Certain transgenic potato plants possessed multiple transgene copies (1 to 5 copies). Three events with one copy of the *StAPI5* transgene were chosen for further analysis. Additionally, pRI-AN201-*StAPI5* plasmid DNA was employed as a positive control (Fig. 2g).

### Analysis of endogenous and transgene expression in transgenic plants

Real-time PCR was also utilized to evaluate *StAPI5* transcriptional expression in transgenic and non-transgenic potato plants. The designed primers had a single melting curve peak, indicating reasonable amplification specificity. Furthermore, high primer efficiency was observed for the *StAPI5* gene (96.0%), the coat protein gene of PVY (101%), and PVA (95.1%) primers, as well as the internal control gene (98.1%).

**Fig. 3:**
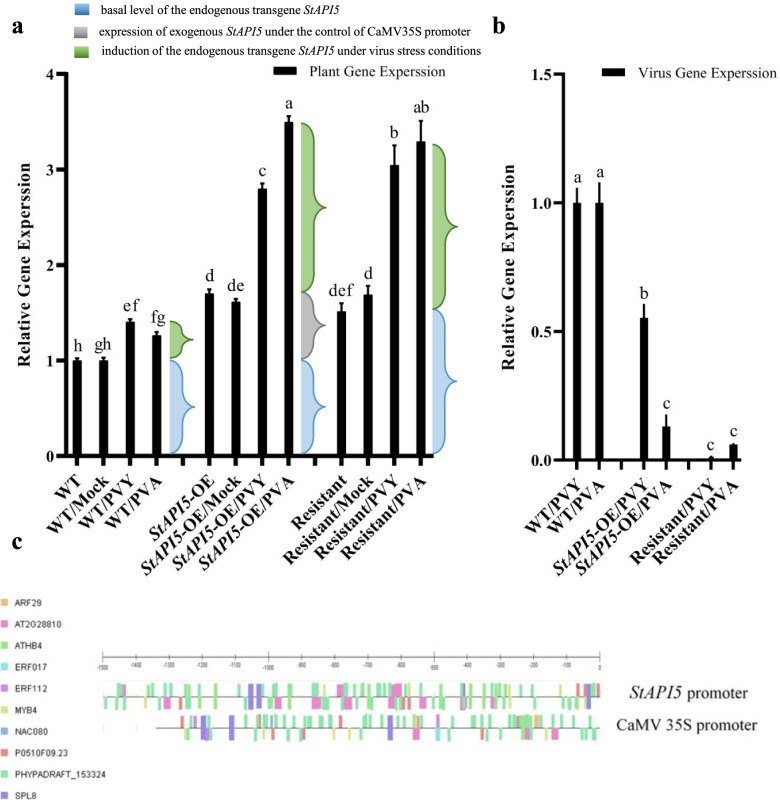
Different patterns of expression obtained through real-time PCR for the *StAPI5* gene and the virus coat protein gene of potato virus Y (PVY) and potato virus A (PVA) in the transgenic and non-transgenic potato plants. **a** Relative gene expression of *StAPI5* at the same developmental stages and tissues. Blue braces, basal levels of the endogenous transgene *StAPI5*; green braces, induction of the endogenous transgene *StAPI5* under virus stress conditions, and gray brace, the expression of exogenous *StAPI5* under the control of the CaMV 35S promoter in the transgenic plants. **b** Expression of coat protein gene (PVY and PVA) in *StAPI5*-OE transgenic potato plants compared to the healthy control ones. WT: wild-type, Resistant: resistant, and *StAPI5*-OE: transgenic potato plants. In each case, Mock: mock-inoculated plants, PVY: plants infected with PVY, and PVA: plants infected with PVA. As a positive control of the experiment, the susceptible cv. Desiree to PVY and PVA and as negative controls, Mock-inoculated plants and resistant cv. Degima were used. All the treatments were performed in three biological and technical replicates, respectively. The averages of the fold changes were plotted (± SE) for the three events in each treatment group. Data were calculated based on the 2^–ΔΔCt^ method using the *18S* rRNA gene as an internal control. Columns sharing same letter are not statistically different at the *P* < 0.05 according to the Duncan’s Multiple Range test. **c** Promoter sequences comparison of the endogenous *StAPI5* and CaMV 35S to predict the potential transcription factor binding sites using the CiiiDER tool. The transcription factor binding sites are represented by colored boxes and introduced with the same color on the right. The boxes that are above and below the line represent the sites on positive and negative strands, respectively

There was no significant difference in endogenous potato *StAPI5* expression levels between non-inoculated plants (WT, Resistant, and *StAPI5*-OE) and mock-inoculated plants (WT/Mock, Resistant/Mock, and *StAPI5*-OE/Mock, respectively) (Fig. 3a). The basal level of endogenous *StAPI5* was low in WT plants under normal conditions (Fig. 3a, blue braces), which could be attributed to routine activity in potato plants. Comparing the transcript levels of potato endogenous *StAPI5* genes in WT/PVY and WT/PVA plants to those in WT plants demonstrated that wild-type plants can induce endogenous expression in response to viral infection. The results indicated a 1.404- and 1.269-fold increase in *StAPI5* transcripts in the WT/PVY and WT/PVA plants, respectively, which was similarly low to that observed in WT plants (Fig. 3a, green braces).

There were no statistically significant differences in the levels of *StAPI5* gene expression between *StAPI5*-OE and Resistant plants. The differences in endogenous *StAPI5* gene expression between Resistant/PVY and Resistant plants or between Resistant/PVA and Resistant plants were statistically significant (Fig. 3a, green braces). Additionally, we observed that following PVY and PVA infection, *StAPI5* relative expression was significantly higher in *StAPI5*-OE/PVY and *StAPI5*-OE/PVA plants than in WT/PVY and WT/PVA plants (1.989- and 2.763-fold, respectively).

The mean levels of *StAPI5* expression in *StAPI5*-OE plants (without virus infection) were compared to those in WT plants to account for the increased levels of *StAPI5* expression under the 35S promoter. Compared to WT plants grown under normal conditions, *StAPI5* expression was increased 1.699-fold in *StAPI5*-OE plants, which was deemed significant (Fig. 3a, gray brace).

The effect of virus infection on the induction of endogenous *StAPI5* was determined by comparing the gene expression patterns in *StAPI5*-OE/PVY and *StAPI5*-OE/PVA plants following virus infection to those in *StAPI5*-OE plants. The results indicated that *StAPI5*-OE/PVY and *StAPI5*-OE/PVA might induce endogenous *StAPI5* at a significantly greater rate than *StAPI5*-OE (1.62- and 1.07-fold, respectively) (Fig. 3a, green braces). Following virus infection, endogenous *StAPI5* expression levels (green braces) should be equal in transgenic and wild-type plants. Nonetheless, this amount was significantly greater in *StAPI5*-OE/PVY and *StAPI5*-OE/PVA than in WT/PVY and WT/PVA (2.69- and 6.75-fold, respectively) (Fig. 3a, green braces). As a result, potential TF binding site predictions were made within the endogenous *StAPI5* and CaMV 35S promoter sequences. Our findings indicated that the promoter regions shared some characteristics. The CiiiDER tool was used to predict transcription factor binding sites and the results showed that the endogenous *StAPI5* and CaMV 35S promoter regions share nine TF binding sites. This corresponds to 90% (9 out of 10) of the TF binding sites in the endogenous *StAPI5* promoter and 100% in the CaMV 35S promoter (Fig. 3c).

Real-time PCR analysis was employed to compare the expression level of the *StAPI5* gene in transgenic plants to that of non-transgenic resistance plants to determine whether the *StAPI5*-OE can enhance plant response PVY and PVA. The virus resistance assay revealed no significant difference in the level of *StAPI5* gene expression between *StAPI5*-OE/PVY and Resistant/PVY plants or between *StAPI5*-OE/PVA and Resistant/PVA plants (Fig. 3a). These findings suggest that the induction status of the *StAPI5* gene in *StAPI5*-OE/PVY and *StAPI5*-OE/PVA plants is similar to that in Resistant/PVY and Resistant/PVA plants, respectively. Compared to WT plants, there was no statistically, significant difference in transcript levels between WT/PVY and WT/PVA plants. Additionally, both transgenic and resistant cultivars exhibit a similar response to virus infection.

### Virus resistance assay of the transgenic plants

To determine whether overexpression of *StAPI5* conferred resistance to PVY and PVA, we compared the responses of transgenic plants to each virus to those of corresponding wild-type plants using real-time PCR analysis. *StAPI5* overexpression resulted in a significant decrease in PVY and PVA coat protein (CP) gene expression, by up to 0.55- and 0.13-fold in *StAPI5*-OE/PVY and *StAPI5*-OE/PVA plants, respectively compared to WT/PVY and WT/PVA (Fig. 3b). The overexpression level of *StAPI5* displayed a strong significant negative correlation with the viral accumulation level (r = − 0.948, *P* < 0.01) (Additional file 4: Fig. S4). Moreover, no significant differences in the level of viral coat protein gene expression were observed between resistant and transgenic plants in inoculated leaves following viral infections. In other words, the viral resistances of the infected transgenic plants were highly similar to those of the resistant infected cultivars.

**Fig. 4:**
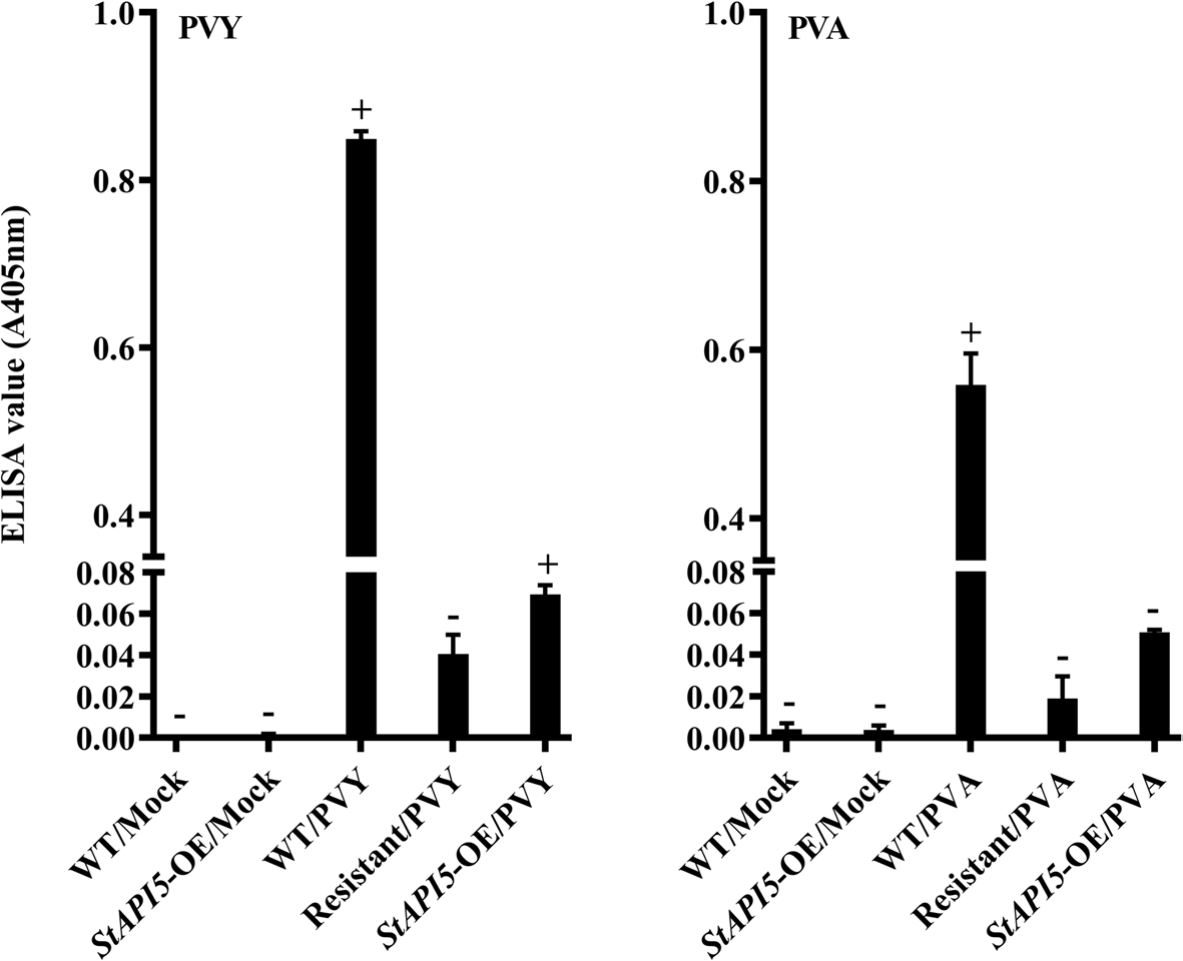
ELISA plate readings of *StAPI5* overexpressing transgenic potato plants to detection of viral accumulation potato virus A (PVA) and potato virus Y (PVY) coat proteins at 21 days after inoculation. WT: wild-type, Resistant: resistant, and *StAPI5*-OE: transgenic potato plants. In each case, Mock: mock-inoculated plants, PVY: plants infected with PVY, and PVA: plants infected with PVA. The susceptible cv. Desiree (Wild-type) and resistant cv. Degima to PVY and PVA were used as control plants. Values represent mean for the biological replicates (*n* = 3, ± SE) with three replicates each. Average of three biological replicates is plotted

Additional analyses were performed to compare the virus CP accumulation in control and transgenic plants grown under normal conditions using an ELISA, 21 days after inoculation (Fig. 4). Simultaneously, the obtained results corroborated the real-time PCR results. The result was a decrease in PVY and PVA CP accumulation in the leaves of inoculated transgenic plants (*StAPI5*-OE/PVY and *StAPI5*-OE/PVA, respectively), compared to the virus-infected wild-type plants (Fig. 4). On the other hand, PVY and PVA CP accumulation were more significant in the leaves of positive control plants (WT/PVY and WT/PVA) compared to the virus-infected transgenic plants. Both viruses accumulated no CP in Mock/PVY and Mock/PVA plants, which served as negative controls.

### Evaluation of morphological characteristics

**Fig. 5:**
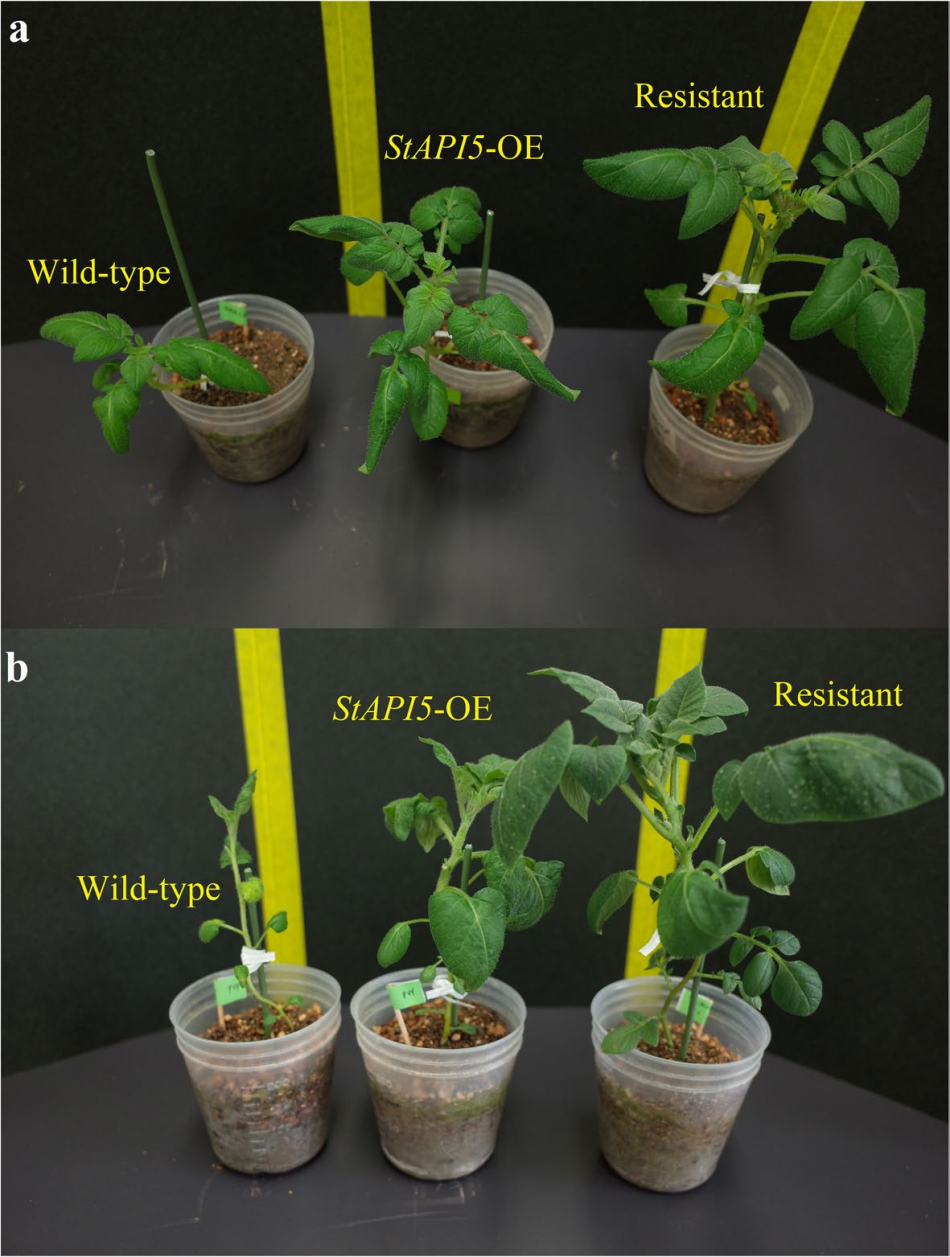
Improved plant growth rate of transgenic and non-transgenic potato plants after **a** PVA and **b** PVY inoculation, respectively at the same developmental stages. The susceptible cv. Desiree (Wild-type) and resistant cv. Degima to PVY and PVA were used as control plants

The growth phenotypes of control and transgenic plants were compared to determine whether overexpression of the *StAPI5* gene has a detrimental effect on plant growth and development processes. The findings indicated that overexpression of the *StAPI5* gene had no discernible effect on plant growth and development (Additional file 5: Fig. S5). When WT and *StAPI5*-OE plants were exposed to virus stress conditions, the leaves of the WT plants exhibited chlorosis, decreased leaf size, and decreased growth, whereas the transgenic plants’ leaves exhibited only a slight effect. PVY also induced more severe symptoms in WT plants than PVA. The difference between *StAPI5*-OE/PVY and *StAPI5*-OE/PVA plants, as well as between WT/PVY and WT/PVA plants, was visible. *StAPI5*-OE/PVY and *StAPI5*-OE/PVA plants grew faster and were healthier than WT/PVY and WT/PVA plants (Fig. 5).

Additionally, growth parameters were measured to determine whether there were any significant differences between transgenic lines and the WT under non-stressed conditions (Additional file 6: Table S1). However, significant differences in plant growth traits were observed between *StAPI5*-OE and WT plants under virus stress conditions. Compared to control plants, transgenic plants (*StAPI5*-OE/PVY and *StAPI5*-OE/PVA) infected with the virus had a greater leaf area and a faster growth rate (a longer stem length, stem diameter, and node number), and a higher fresh/dry weight (Additional file 6: Table S1). Internode length was considerably shorter in virus-stressed transgenic plants (*StAPI5*-OE/PVY and *StAPI5*-OE/PVA) than in WT/PVY and WT/PVA plants. Moreover, the results indicated a significant reduction in plant growth-related characteristics in WT plants when compared to Resistant/PVY and Resistant/PVA plants (Additional file 6: Table S1).

### Effect of stress on photosynthesis and gas exchange variables

We compared changes in photosynthetic responses between transgenic and control plants that had been inoculated with the virus. The results revealed that almost all growth and physiological traits of *StAPI5*-OE/PVY and *StAPI5*-OE/PVA plants were enhanced compared to WT/PVY and WT/PVA plants (Additional file 6: Table S1). There were no significant differences in gas-exchange and photosynthesis parameters between infected resistant and transgenic plants (Additional file 6: Table S1). The Fv/Fm rate, stomatal conductance and transpiration, net photosynthetic rate, intercellular carbon dioxide concentration, and leaf temperature were all increased in transgenic potato plants when infected with the virus. The *F*_*V*_/*F*_*M*_ of WT/PVY and WT/PVA plants decreased significantly compared to *StAPI5*-OE/PVY and *StAPI5*-OE/PVA plants, indicating that *StAPI5*-OE/PVY and *StAPI5*-OE/PVA plants withstood the stress conditions significantly better than WT/PVY and WT/PVA plants. As expected, the *F’v*/*F’m* and qN parameters of WT/PVY and WT/PVA plants were higher than those of *StAPI5*-OE/PVY and *StAPI5*-OE/PVA plants under stress conditions.

**Fig. 6:**
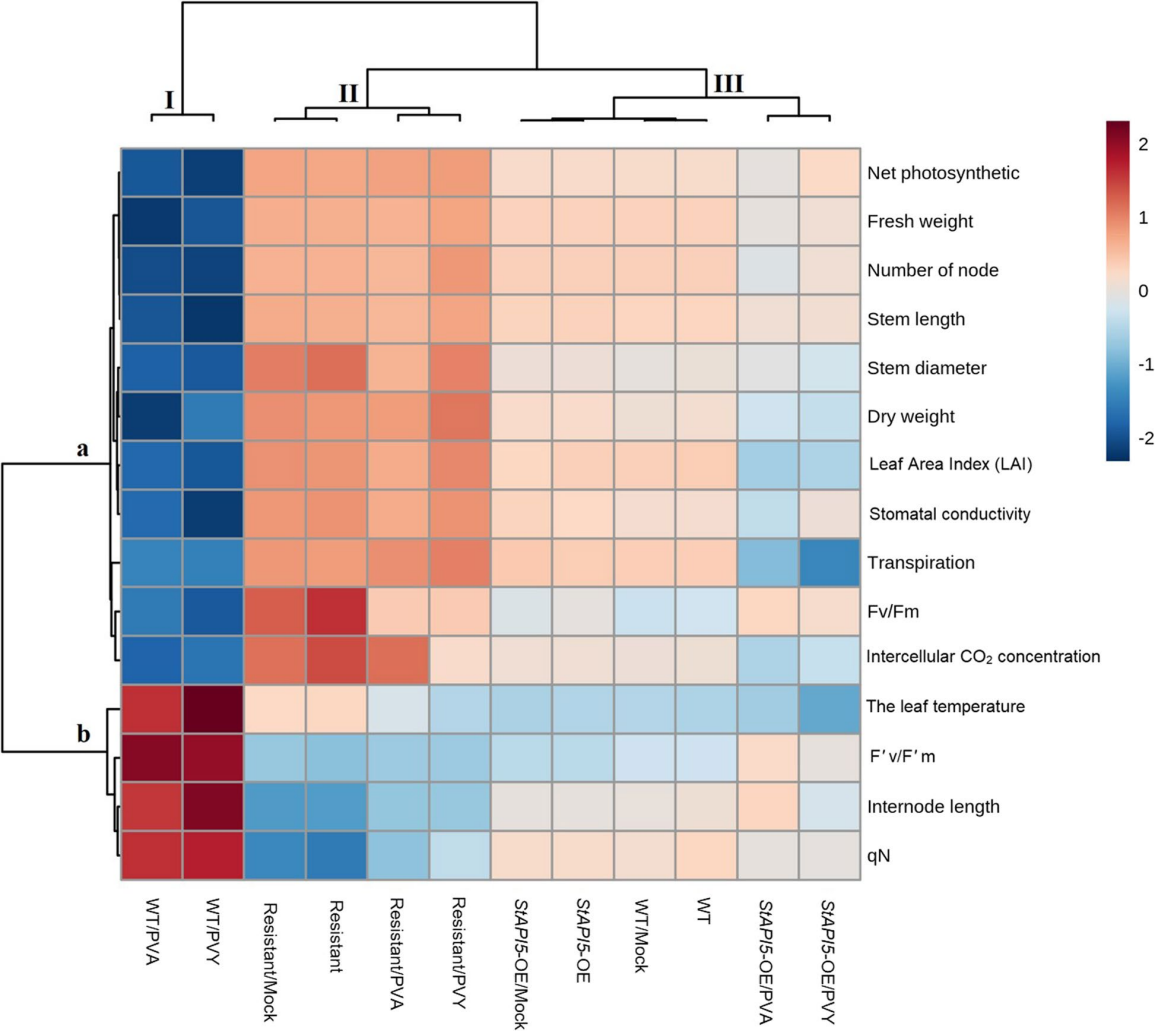
Heat map representation for some measured morphological and physiological parameters of transgenic and non-transgenic potato plants at the same developmental stages 21 days post inoculation with virus. WT: wild-type, Resistant: resistant, and *StAPI5*-OE: transgenic potato plants. In each case, Mock: mock-inoculated plants, PVY: plants infected with PVY, and PVA: plants infected with PVA. The susceptible cv. Desiree and resistant cv. Degima to PVY and PVA were used as control plants. The colors closest to red indicate that the value of each parameter is higher than its average value and blue is lower than its average value

The virus responses of transgenic and control plants were also evaluated physiologically and morphologically using heat map hierarchical clustering (Fig. 6). The results of this analysis assigned inoculated wild-type plants (WT/PVY and WT/PVA) to a single branch (I), effectively separating them from the rest of the plants (transgenic and non-transgenic plants). This means that PVY and PVA had a detrimental effect on the performance of WT plants. In comparison to inoculated wild-type plants (WT/PVY and WT/PVA), inoculated or non-inoculated resistant plants (Resistant, Resistant/Mock, Resistant/PVY, and Resistant/PVA) formed an isolated branch (II). In the other words, inoculated wild-type plants exhibited stronger stress responses than inoculated or non-inoculated resistant plants across all physiological and morphological traits.

Furthermore, non-inoculated wild-type plants and inoculated or non-inoculated transgenic plants (*StAPI5*-OE, *StAPI5*-OE/Mock, *StAPI5*-OE/PVY, and *StAPI5*-OE/PVA) were clustered in an isolated branch (III) (Fig. 6). This means that virus stress cannot have a detrimental effect on the performance of inoculated transgenic plants. Stress treatment plants were established as a distinct sub-branch within both branches II and III.

## Discussion

Developing new disease-resistant plant species is vital to protect plants against various pathogens. Enhancing the plant innate immune response of plant is critical for significantly reducing resistance breakage by new virus strains. Plant cells begin reprogramming the various signaling events from stimulus sensing to the final response to environmental challenges (biotic and abiotic stresses). Numerous studies indicate that numerous genes involved in defense responses to biotic and abiotic stresses may induce or antagonize one another. Thus, overexpression of specific genes can decrease or increase susceptibility to other stresses in plants [33–35].

Additionally, multiple stress-related pathways may activate a single signaling mechanism [36]. It is critical to identify the primary factor affecting both biotic and abiotic stress response pathways to open up new avenues for developing plant tolerance to a wide variety of concurrent stresses. Selecting host candidate genes related to resistance against various stresses significant in breeding programs.

Our previous study used publicly available microarray data, such as GEO and Arrayexpress, which identified and classified a set of stress-responsive genes. These candidate genes were critical for potato plant resistance to damage-causing viruses (PVY, PVA, and PLRV) and a variety of other significant biotic and abiotic stresses. Notably, we selected genes with a significant effect in resistant potato plants exposed to virus stress conditions, but not in susceptible plants, using combined microarray meta-analysis results. As a result, the significant increase in expression of selected genes can be attributed to the basal defense responses of plants to stresses. Additionally, the results indicated that resistant cultivars expressed significantly more PIs than sensitive cultivars under virus stress conditions, indicating their vital roles in plant defense against viruses [30]. Plants can integrate multiple signaling pathways to enable an appropriate defense response to various stresses [33]; thus, overexpression of stmcq55 may increase resistance to fungi, nematodes, salt, and drought stresses concurrently (Table 1). These genes exhibit no antagonist responses to biotic or abiotic stress. As a result, they may be excellent candidates for successfully engineering potato plant stress resistance.

In response to biotic and abiotic stresses, plants recognize pathogen elicitors and produce a diverse array of primary and secondary signals (upstream responses) that activate various plant protector and defense genes. Following that, defense genes protect against pathogen invasion by producing glutathione S-transferases, proteinase inhibitors, peroxidases, PR proteins, and hydrolytic enzymes (downstream responses) [37]. Defense-related genes, such as stmcq55 (Fig. 1) as a type of the pathogenesis-related [PR] protein [PR-6], which encodes proteinase inhibitors, are generally involved in downstream responses [13, 16, 31, 32]. The study of plant defense responses to pathogens has demonstrated that altering or overexpressing of a single signaling pathway gene, which regulates many defense-responsive genes, can confer resistance to a wide variety of pathogens [15, 38]. This approach, however, may result in growth repression and yield loss as a result of constitutive overexpression of a substantial number of genes at the same time [15]. Thus, careful consideration should be given to selecting signaling pathway genes.

The genes that activate partial pathways, such as downstream response-related genes, are excellent candidates for engineering resistance. Numerous genetic studies have shown that overexpression of stress-responsive genes can increase a plants’ resistance to various stresses [39, 40]. As previously stated, transgenic plants grew and developed were significantly faster than wild-type plants when PVY and PVA treatments were used (Additional file 6: Table S1, Fig. 5). This contrasts with previous research in which transgenic tobacco, potato, and rice plants expressing PIs as a defense mechanism against potyviruses and bacterial phytopathogens were used without observing such adverse effects [22, 41, 42]. However, overexpression of a PI in transgenic tobacco or Arabidopsis plants has not been shown to improve tolerance to salinity or osmotic stresses.

Despite this, it increased yield, seed germination, root length, root-shoot (root/shoot or shoot/root) ratio, total chlorophyll content and proline content when exposed to salinity and osmotic stress [41, 43]. Several beneficial pleiotropic effects have been observed in transgenic tobacco plants expressing cysteine proteinase inhibitors against potyviruses compared to control plants, including increased growth rate, seed production, and flowering several days earlier [22]. Thus, in addition to conferring resistance to biotic and abiotic stresses, overexpression of PI genes promotes growth and yield enhancement under stressed conditions.

The most prominent problem in real-time PCR analysis is the selection of a stable reference gene for a precise gene expression study. The result of one study represented that *18S* rRNA is one of the most stable reference genes in cereal plants under conditions of viral infections, giving very good statistical reliability [44]. In addition, *18S* rRNA displayed much higher detection sensitivity than most of the reference genes. In this study, we employed *18S* rRNA as a reference gene for real-time PCR analysis. The current study’s findings indicate that *StAPI5* was significantly induced in transgenic (*StAPI5*-OE/PVY and *StAPI5*-OE/PVA) or resistant plants (Resistant/PVY and Resistant /PVA). In comparison, corresponding susceptible wild-type plants (WT/PVY and WT/PVA) cannot significantly induce the *StAPI5* gene in response to virus infection. Thus, transgenic potato plants overexpressing the *StAPI5* transgene (*StAPI5*-OE/PVY and *StAPI5*-OE/PVA) enhance not only the ability of endogenous *StAPI5* to be adequately induced but also virus resistance to PVY and PVA, respectively. Additionally, it was demonstrated that when transgenic potatoes were challenged with the virus, endogenous *StAPI5* gene expression was dramatically increased, whereas wild-type plants were unable to induce the *StAPI5* gene in response to virus infections significantly. These findings suggest that the CaMV 35S promoter can benefit the endogenous *StAPI5* promoter’s activity. Moreover, it has been demonstrated in transgenic tobacco lines that strong promoters can have a synergistic effect on weak promoters when they share TFs- binding sites [45]. The analysis of the CaMV 35S and *StAPI5* promoters revealed that both shared a large number of TF binding sites (Fig. 3c). The same effect (enhancer activity) was observed in potato when the endogenous *STSAR1A* promoter was induced by the 35S promoter which shared ten TF binding sites [46].

Numerous models have been proposed to explain enhancer activity in various promoters, including the presence of an increased number of similar cis-elements in enhancer promoters which results in a higher local frequency of TF, increasing the possibility of binding to the endogenous promoter in the surrounding area [45]. Protein–protein interaction, protein modification, chromatin structure rearrangement and nucleosome rearrangement have been suggested as possible mechanisms [47–50]. As the majority of the TF binding sites in the endogenous *StAPI5* and CaMV 35S promoter regions are identical, the effect of a higher local TF frequency may be the most probable explanation for endogenous *StAPI5* gene induction.

As indicated by the heat maps, virus-affected wild-type (WT/PVY and WT/PVA) plants were significantly more negatively affected than uninfected wild-type (WT) plants on all traits and thus form a distinct clade. Furthermore, these findings indicate no discernible differences in the measured parameters of infected transgenic (*StAPI5*-OE/PVY and *StAPI5*-OE/PVA) plants and non-infected wild-type plants (WT) (Fig. 6, Additional file 6: Table S1). These results suggest that transgenic plants can be considered PVY and PVA resistant due to their ability to maintain a high level of performance across a range of virus stress conditions for all parameters studied (morphological and physiological traits). Moreover, under non-stress conditions, the transgenic plants (*StAPI5*-OE) and the wild-type plants (WT) cluster together (Additional file 6: Table S1). Additionally, these results demonstrated that transgenic plants overexpressing *StAPI5* did not exhibit any detrimental changes in growth and development. As a result, these plants can be used to develop future breeding strategies. Transgenic and resistant plants inoculated with viruses also grew normally, which exhibited the same levels of virus responses.

The heatmap results also indicated that infected WT plants (WT/PVY and WT/PVA) exhibited significant adverse effects on internode length, qN, F’v/F’m, and leaf temperature. By contrast, the overall trend of our heatmap results indicated that these traits, as well as chloroplast function and photosynthesis, were less affected by virus infection in transgenic and resistant potato plants, correlating with our real-time PCR results (Fig. 6). These factors have been used to identify and select the desired genes as molecular markers. Harvesting enough light energy to sustain growth may account for the apparent benefit of longer internodes on plant performance [51, 52]. According to several studies, increased temperatures decreased leaf area and net photosynthesis, resulting in decreased potato yield and growth performance [53, 54]. FV’/FM’ has been positively correlated with plant photosynthesis and total yield efficiency in potato plants [55]. Thus, in the current study, improvements in the leaf temperature, qN, internode length, and F’v/F’m are critical factors that contribute significantly to the improved plant growth performance of *StAPI5*-OE/PVY and *StAPI5*-OE/PVA plants, respectively when compared to WT/PVY and WT/PVA.

Various environmental stresses have a detrimental effect on crop growth and yields. Virus infection has a detrimental negative effect on plant growth-related traits, such as the number and area of leaves in wild-type plants. These adverse effects can decrease photosynthesis activity, which provides energy for growth and defense against pathogens. This decrease in photosynthesis activity is closely associated with yield losses and product quality degradation [56]. Reduced levels of these plant-related factors resulted in shorter plants, fewer leaves, slender stems, and decreased biomass in virus-affected plants compared to uninfected plants [57]. Photosynthesis is a vital process that is significantly impaired during viral infection [58]. We measured the maximum quantum efficiency of photosystem II (Fv/Fm) to determine the changes in fluorescence parameters under control and virus stress conditions.

Recent reports indicate that Fv/Fm is a highly effective and widely used parameter for examining the photosynthetic efficiency of various crop plants exposed to the virus and other stresses [59–61]. The *StAPI5*-OE/PVY and *StAPI5*-OE/PVA plants in this study exhibited higher Fv/Fm values than the WT/PVY and WT/PVA plants, respectively, under virus stress conditions. These findings suggest that when transgenic plants are inoculated with viruses, they maintain a higher optimal health level than wild-type plants. In Arabidopsis, an apoplastic aspartic protease overexpression results in dwarfing and resistance to virulent *Pseudomonas syringae*. This resistance developed due to pathogen-induced induction of the protease inhibitor pepstatin [62]. A review of the findings indicates that PIs are essential for manipulation as a defense mechanism against biotic and abiotic stresses [63]. PIs are induced locally and systemically in response to pathogen attacks, supporting the notion that they contribute to plant disease resistance improvement [64].

*StAPI5* is a member of plant protease inhibitors (PIs), which are frequently induced in response to pathogens and received considerable attention in recent years due to their importance in host defense mechanisms against viral and non-viral pathogens [65, 66]. Furthermore, PIs play a role in regulating plant programmed cell death [67]. Moreover, it was demonstrated that introducing a single PI gene into the plant makes it possible to protect it against pathogens [42]. Our findings indicate that overexpressing the *StAPI5* gene transgenic potato plants exhibit decreased virus accumulation and increased resistance to PVY and PVA. Potyvirus RNAs encode a large polyprotein that is processed proteolytically in infected cells by three potyvirus-encoded proteases: P1, helper component proteinase (HC-Pro), and nuclear inclusion proteinase (NIa-Pro). The virus used this process to generate at least ten mature individual viral proteins [68].

Because protease activity is required to replicate potyviruses, PIs may be used to inhibit viral replication. For instance, constitutive expression of a rice cysteine proteinase inhibitor in tobacco plants may enhance resistance by reducing PVY and tobacco etch virus (TEV) accumulation in infected cells [22]. The strategy of using PIs as a defense mechanism can be used against viruses that rely on protease activity for genome expression and replication. Given that overexpression of the *StAPI5* gene reduced virus accumulation in transgenic potato plants in our study, at least one of the three potyvirus-encoded proteases may be of the aspartic type. As a result, one method for achieving such resistant transgenic plants is to use the corresponding proteinase inhibitor to inhibit viral replication in a specific plant virus.

## Conclusion

*StAPI5* is significantly induced during virus infection in resistant cultivars, according to our previous research on potato expression profiles. In conclusion, in the present study, we successfully generated transgenic potato plants that express *StAPI5* under the control of the constitutive CaMV 35S promoter. Our findings establish a critical role for *StAPI5* in the response of potato plants to virus stresses (PVY and PVA). Increased resistance to virus stresses was observed in susceptible cultivars when *StAPI5* was overexpressed. Additionally, the results compared several morphological characteristics of transgenic and non-transgenic plants and established that overexpression of *StAPI5* in transgenic potato plants has no detrimental effect on transgenic plant growth. Based on the similarity in observed physiological and morphological responses to virus stresses in resistant and transgenic plants, we can deduce that the same regulatory and subsequent physiological programming was used to enhance these plants’ ability to fight viruses. The improved growth performance of transgenic plants in the presence of virus stresses demonstrates that the *StAPI5* gene is critical for increasing plant resistance to virus stresses.

These findings demonstrate that selecting of *StAPI5* based on data analysis is an effective strategy for improving plant performance across multiple traits and resistance to viruses. We anticipate that the transgenic potato plants used in this study may be more resistant to stress (resistant to fungi, nematode, salt, and drought stresses simultaneously based on the heatmap results). As a result, additional research is required to determine the role of *StAPI5* in resistance to other stresses. We must emphasize that the results of our research on transgenic Desiree potato plants, may not be replicated when *StAPI5* is overexpressed in other plants or even other potato cultivars. Thus, further research and evaluation of the *StAPI5*’s function and role in other plants are required. Increased resistance to PVY and PVA was observed in transgenic potato plants overexpressing the *StAPI5* gene. However, studies are warranted to develop transgenic potato plants that are more resistant to other potato plant essential viruses such as PLRV, PVX, and PVS. Our study results add to the growing body of knowledge regarding *StAPI5*’s defense mechanism and suggest novel directions for developing new virus-resistant plant species.

## Material and methods

### Plant materials and growth conditions of potato plants

The potato (*Solanum tuberosum* L.) seed tubers of the susceptible cv. Desiree and resistant cv. Degima to PVY and PVA, were grown at a temperature of 22/18 °C, under light/dark cycles in condition 16 h of light and 8 h of dark in the greenhouse conditions University of Hokkaido, Sapporo, Japan. The potato seed tubers were purchased from TAKII & CO., LTD which were produced in Nagasaki Prefecture. For plant propagation, 3–4 weeks after germination, nodes were transferred to glass tubes containing 15 ml liquid MS [69] basal medium (Murashige and Skoog Plant Salt Mixture, Wao, Osaka, Japan). The basal medium was supplemented with vitamins and sucrose, as previously described [30]. The propagated plants were placed under the growth chamber (light/dark regime of 16/8 h at 25/22 °C, relative humidity of 60%). For plant transformation, the internode pieces (4–6 mm) of sterilized stem explants were used.

### Virus isolates and sources

The infectious virus clones were used as a source of PVY (the ordinary strain of PVY, PVY^O^) and PVA (with the accession number of MAFF307028 from the NARO Genebank; https://www.gene.affrc.go.jp/databases-micro_search_detail.php?maff=307028) and were maintained on tobacco (*Nicotiana tabacum*) cv. benthamiana by mechanical inoculation. For maintenance of virus, 1 g of infected leaves was macerated well with 1 mL of phosphate buffer (0.5 M, pH 7.0) and used to mechanically rub on carborundum pre-dusted of 3–4 leaf stage. Leaves were rinsed after inoculation, and the inoculated plants were maintained in the controlled conditions of the growth room at 16-h light/8-h dark and 25 °C. The upper contaminated leaves of tobacco plants were collected at seven days post-inoculation (d.p.i) and used for rub-inoculation of transgenic potato plants.

### Expression cassette

The full-length coding sequence (CDS) of the gene encoding aspartic protease inhibitor 5 (*StAPI5*) from potato (*Solanum tuberosum* L. cv. Desiree) was amplified by polymerase chain reaction (PCR) with two gene-specific primers: *API5*-F and *API5*-R (Additional file 7: Table S2) and Max Taq DNA polymerase (Vivantis Technologies, Malaysia) according to the manufacturer’s instructions. The *StAPI5* gene was cloned into the plant expression vector pRI201-AN (Takara Bio., Tokyo, Japan). The resulting recombinant plasmid, pRI201-*StAPI5*, under the control of cauliflower mosaic virus 35S promoter (CaMV35S) and an HSP (heat shock protein) terminator was introduced into potato plants. Digestion of the pTZ57 vector containing the ampicillin resistance marker and CDS of the *StAPI5* gene was done with restriction enzyme pairs *Sal*I/*Xba*I. The pRI201-*StAPI5* overexpression vector was then achieved by ligation of the digested template fragment with the *Sal*I/*Xba*I digested destination vector (pRI201-AN). The resulting plasmid vectors were used to transform *Agrobacterium tumefaciens* strain LBA4404. Subsequently, Agrobacterium cells were employed to introduce *StAPI5* expression cassette into the internode segments of potato plants cv. Desiree. The transformation procedure was performed as previously described [29, 30]. After tan days of growth, the rooted transformants were transferred into the plastic pots in a soil mix containing peat/perlite/vermiculite (1/1/1, v/v/v). The transformants were placed in a controlled environment growth room at a light intensity of 100 W m^− 2^ and 25 and 22 °C during 16-h days and 8-h nights. Transformation efficiency (percentage of explants producing at least one shoot) was evaluated in a selective regeneration medium with kanamycin and cefotaxime antibiotics. As a control medium, the regeneration efficiency was used and assessed in an unselective regeneration medium without antibiotics.

### Selection of transformed plants and analysis of the transgenic plants

#### Genomic verification

DNA was isolated from the transgenic potato leaves following Plant DNAzol reagent (Invitrogen, USA), according to the manufacturer’s instructions. The resulting transformants were verified by PCR using the forward primer specific for 35S promoter of the vector (35S-F) and gene-specific reverse primer (*API5*-R). All primers used in this study are shown in Additional file 7: Table S2. Genomic DNA was isolated from untransformed potato plant and pRI201 plasmids containing the transgene (*StAPI5*) were used as negative and positive controls, respectively.

#### Southern-blot analysis

Positive putative transformants and control (non-transformed) plants detected by PCR were subjected to demonstrate transgene integration of transgene by Southern-blot analysis. One-month-old transgenic plants grown in the controlled conditions of the growth room were investigated. Genomic DNA was extracted from the leaves of three-week-old plants according to the Plant DNAzol reagent. Isolated DNA was purified by RNase treatment, quantified by UV spectrophotometry, and digested with *EcoR*I. For making probes, plasmid-DNA containing the full-length cDNAs of *StAPI5* (pRI201- *StAPI5*) served as PCR-templates using KOD FX Neo DNA polymerase (Toyobo, Osaka, Japan). To synthesis the transcript, the gene-specific primer (downstream) contained an artificially introduced T7-promoter at its 5′-end were used with the gene-specific primer (upstream, *API5*-F) (Additional file 7: Table S2). Quantification of purified PCR-products was performed using a nanodrop spectrophotometer. 200 ng of PCR product were subsequently used as template DNA for generating DIG-labeled RNA probes by PCR according to the in vitro transcription (Roche, Germany). Southern blot analysis was performed according to Sambrook and Russel method [70].

#### Detection of viral infection

For sap inoculation, virus-infected leaves (1 g) of *N. tabacum* var. benthamiana were collected and ground in 1 ml of 0.1 M phosphate buffer. The fresh sap was used to inoculate the transgenic, resistant, and wild-type plants with similar viral doses. One week after virus inoculation (PVY and PVA separately), the target gene expression patterns (*StAPI5*) in each plant were detected. Real-time PCR was done using virus primers from the viruses’ coat protein gene region and gene-specific primers, generating PCR fragments lower than 200 bp (Additional file 7: Table S2). All real-time PCR data were collected from three biological replicates and all cDNA samples were assayed in three technical replicates. The data were obtained from the average of the biological replicates. Total RNA was extracted from plant samples using the Plant Total RNA purification kit (cat#TR02–150, Molecular Biology Tools), following the manufacturer’s protocol. The isolated RNA was treated with RNase-free DNaseI (Takara, Japan) to remove any contamination with genomic DNA. The quantity of the isolated RNA was measured using a Nanodrop spectrophotometer. The cDNA was produced from 1 μg of total RNA using both random hexamer and oligo (dT(primers and also MMuLV reverse transcriptase enzyme (Fermentas) by following the manufacturer’s instructions. Amplification of the target genes and viral genes was performed using an AriaMx Real-time PCR system (Agilent Technologies, Japan). 1 μl template cDNA (diluted 1:20) was run with iQ SYBR Green Supermix (Bio-Rad) (final volume = 20 μl). The following thermal protocol was used: 3 min hot-start at 95 °C, followed by 40 cycles of amplification at 95 °C for 5 s, 60 °C for 10 s, 72 °C for 20 s. subsequently, melting curves were generated as follows: 95 °C for 30 s, 65 °C for 30 s, 95 °C for 30 s. The relative expression levels of the *StAPI5* gene and the coat protein gene region of PVY and PVA were calculated using the *18S* ribosomal RNA (X67238) gene as the used reference/an internal control.

The relative changes in gene expression were determined according to the formula 2^-ΔΔCt^ [71, 72]. The melt-curve analysis was performed to check real-time PCR reactions for primer-dimer artifacts and verify the primers’ specificity. Standard curves were generated using the serial dilutions of cDNA samples ranging from 10^− 1^ to 10^− 5^ to confirm the primer efficiency. Control plants were Resistant, *StAPI5*-OE, and WT (non-inoculated potato plant); Resistant/Mock, *StAPI5*-OE/Mock, and WT/Mock (mock-inoculated potato plant treated only with inoculation buffer), and Resistant/PVY, Resistant/PVA, WT/PVY, and WT/PVA (virus-infected control plants). All plants were grown at similar stages of growth and under the same growth conditions.

#### Enzyme linked immunosorbent assay (ELISA)

Evaluation of the virus infection in transgenic and non-transgenic plants was carried out by the direct double antibody sandwich form of ELISA (DAS-ELISA) procedure [73]. Equal amounts of fresh leaf tissue were sampled from the same areas and on the same day. Each treatment was tested separately 3 weeks after viral inoculation for virus susceptibility using anti-CP polyclonal antibody (Japan Plant Protection Association) to detect PVY and rabbit polyclonal antibody (https://orders.agdia.com/agdia-set-pva-alkphos-sra-60000) to detect PVA. Each well’s optical density was read at 405 nm with a plate reader (ARVO MX 1420 Multilabel Counter, Perkin-Elmer). The absorbance values were corrected by subtracting the average triplicate absorbance readings for each sample and the average buffer blanks triplicate readings. Virus-infected samples were those with the mean absorbance values higher than R (the mean ± standard deviation for triplicate independent of the negative control samples) [74]. Blank, mock-inoculated (WT/Mock and *StAPI5*-OE/Mock), and virus-resistant potato cv. Degima inoculated with PVY and PVA separately (Resistant/PVY and Resistant/PVA) plants were grown as negative controls while the virus-inoculated potato wild-type (WT/PVY and WT/PVA) as positive controls.

### Determination of gas exchange and chlorophyll fluorescence parameters

Both control and transgenic potato plants infected with PVY and PVA were used to determine the possible effects of the virus infection on the Gas exchange and photosynthetic parameters. Three to nine plants for each treatment were assayed, and for all treatments, two leaves were harvested at the same positions of each plant. A Portable Photosynthesis System Ver. 6 (LI-6400XT, LI-COR Inc., U.S., Canada) was used to measure the gas exchange parameters. Analyses of the various photosynthetic parameters were conducted on the 3rd leaf plants counting from top downward on 8-week-old plants between 11 A.M and 3 P.M. local time on the same day. Replications were three plants per treatment to perform measurements. For simultaneous assessment of the major fluorescence parameters of chlorophyll (Chl), a portable modulated fluorometer (PAM-2000, Walz, Effeltrich) was used at the same time and position on the leaf where the gas exchange was evaluated.

### Evaluation of physiological and growth-related parameters

After gas exchange and chlorophyll fluorescence measurements, some morphological characteristics, including the number of node/plant, stem length (cm), internode length (cm), and stem diameter (mm), were evaluated on the same day (8-week-old plants). The newly full expanded leaves (the fourth one from the top of the shoots) at the same time (3 seedlings per treatment) were cut and measured the Leaf area index (LAI) (m^2^ plant^− 1^). Also, in each plant, all leaves were cut and then weighed to calculate the fresh weight (g plant^− 1^). The leaves were then oven-dried at 50 °C until constant weight to evaluate the dry weight (g plant^− 1^).

### Data analysis

Visualization of the results and regulatory pathways during plant interactions with the virus was performed using MapMan software (V.3.5.1R2).

Oligo Ver. 7.54 software is used for designing and analyzing oligonucleotide primers and probe sequences. Real-time PCR and melting curve analysis were assessed and analyzed using the AriaMx Real-Time PCR software. The CiiiDER tool was used for searching transcription factor (TF) binding sites in the promoter regions of endogenous *StAPI5* and CaMV 35S [75].

SAS software (version 8.2; SAS Institute, Cary, NC) is used for the statistical analysis of experimental data. Separation of significant means was also accomplished using Duncan Multiple Range Test [76] at 0.05 significant levels. One-way analysis of variance (ANOVA) was carried out for statistical analyses of multiple experimental groups and comparing transgenic and non-transgenic potato plants’ morphological parameters. The cluster heatmap (based on Ward’s algorithm with Euclidean distance metric) was created through online web-based tools (https://www.metaboanalyst.ca/) for instant visualization of the morphological and physiological data.

## Supplementary Information

**Additional file 1: Figure S1.** The DNA sequencing result of *StAPI5* gene in the expression vector pRI201-AN using vector-specific primer M13 forward by Macrogen company using the Sanger technique.

**Additional file 2: Fig. S2.** PCR analysis of DNA isolated from transgenic plants with CaMV 35 S forward primer (35S-F) and *StAPI5* gene reverse specific primer (*StAPI5*-R) to verify the insertion of the target gene into the potato genome. The expected band length is 754-bp. C: Negative control with non-transgenic potato plant (wild-type); P: positive control PCR reaction with plasmid as template; Lanes no. 1–10: DNA from independent transgenic potato plants and M: 1 kb DNA Ladder.

**Additional file 3: Fig. S3.** Southern blot analysis of the independent transgenic potato plants overexpressing *StAPI5* gene (right) in this image and one other studied gene (left) to confirm the transgene integration into the potato genome and to estimate the transgene copy number. P: the pRI-AN201 binary vector containing the *StAPI5* gene (positive control); lanes 1 and 2: independent transgenic potato plants; C: non-transformed potato (negative control)

**Additional file 4: Fig. S4.** Pearson’s correlation coefficient between the expression level of *StAPI5* and the viral CP accumulation level (significant at *P* < 0.01).

**Additional file 5: Fig. S5.** Comparison of the growth of transgenic (*StAPI5-*OE) and non-transgenic potato plants (Wild-type) at the same developmental stages (8-week-old plants).

**Additional file 6: Table S1.** Values of gas exchange, chlorophyll fluorescence parameters and morphological traits in the resistant cv. Degima, transgenic, and Wilde-type cv. Desiree inoculated with PVA (R/PVA, StAPI5-OE/PVA, and WT/PVA, respectively), PVY (R/PVY, StAPI5-OE/PVY, and WT/PVY), buffer (R/Mock, StAPI5-OE/Mock, and WT/Mock), and without inoculation (R, StAPI5-OE, and WT). The number of replicates in each plant for each treatment was shown as Rep. No. The variables were measured in 21-day-old plants in the growth chamber. Values represent the mean (± SE) of the replicates per treatment. Values within the same row followed by the same letter (s) are not significantly different (Duncan’s multiple range test, *P* < 0.05).

**Additional file 7: Table S2.** Sequences of the gene-specific, coat protein of PVY and PVA, and internal control primers.

## Data Availability

The sequencing data generated in this study for *StAPI5* gene is accessible through NCBI-GenBank accession number MH686153.

## Abbreviations

*API*: Aspartic Protease Inhibitor
PIs: Protease inhibitors
PVY: Potato Virus Y
PVA: Potato Virus A
*F*_*m*_ and *F*_*m*_*′*: The maximal fluorescence in the darkness and light, respectively
*Fv* and *Fv’*: The variable fluorescence in the darkness and light, respectively
*F*_*v*_*/F*_*0*_: The ratio of efficiency of electron donation to photosystem II
*F*_*0*_: Initial fluorescence level (at 50 μs)
*TF*: Transcription factor
*q*_*N*_: The non-photochemical quenching of variable ChlF
WT: Wild-type potato plants cv. Desiree
WT/PVY and WT/PVA: Wild-type potato plants cv. Desiree inoculated with PVY and PVA, respectively
*StAPI5*-OE: Transgenic potato plants cv. Desiree
*StAPI5*-OE/PVY and *StAPI5*-OE/PVA: Transgenic potato plants cv. Desiree inoculated with PVY and PVA, respectively
Resistant: Resistant potato plants cv. Degima
Resistant/PVY and Resistant/PVA: Resistant potato plants cv. Degima inoculated with PVY and PVA, respectively
Resistant/Mock, WT/Mock, and *StAPI5*-OE/Mock: Mock-inoculated samples of resistant potato plants cv. Degima and cv. Desiree for wild-type and transgenic potato plants, respectively
