# Supplementary material for: Aspartic protease inhibitor enhances resistance to potato virus Y and A in transgenic potato plants
Source: BMC Plant Biol. 2022 May 12;22:241. doi: 10.1186/s12870-022-03596-8 (PMC9097181; doi:10.1186/s12870-022-03596-8)

## Slide 1
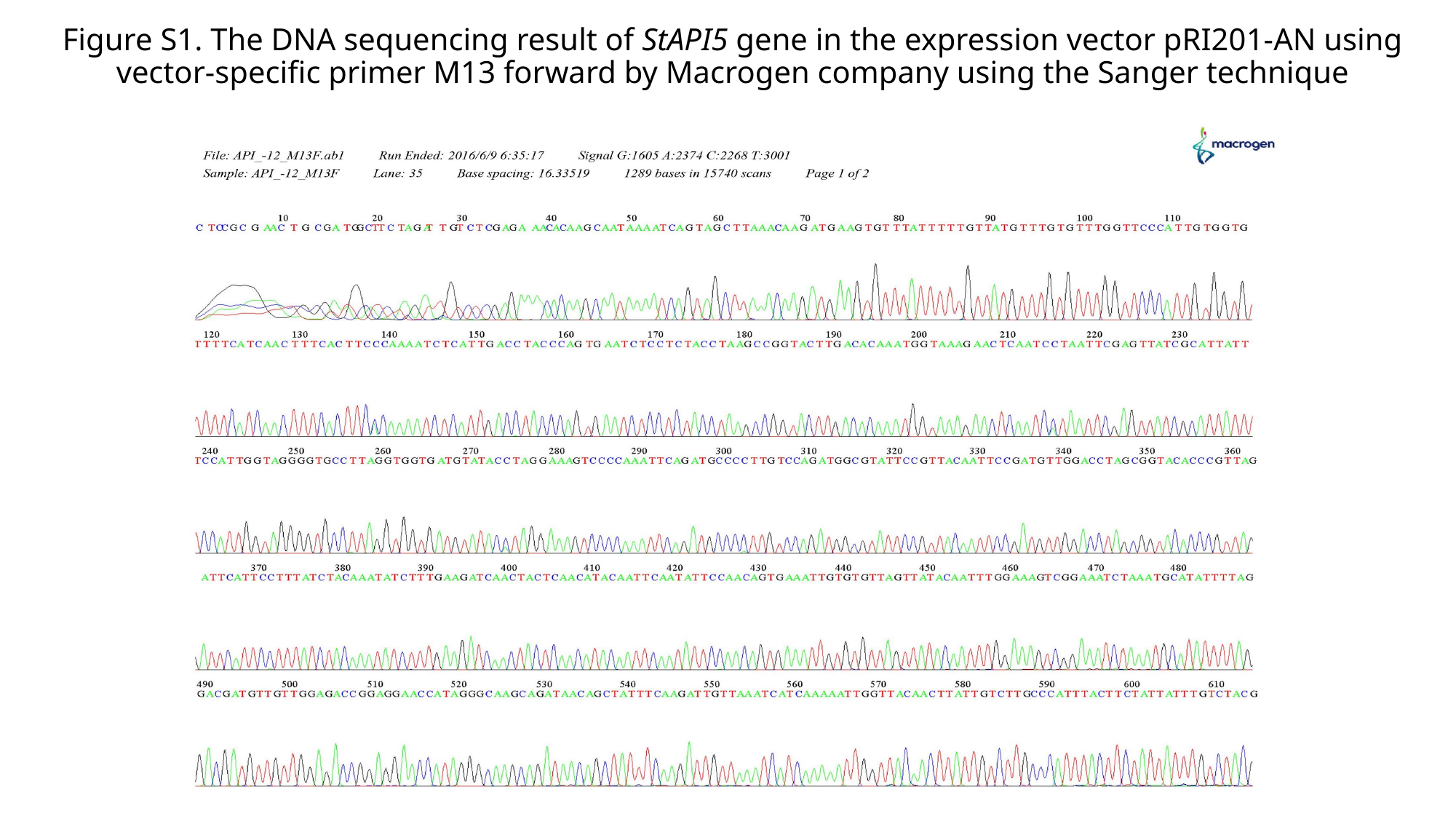

# Figure S1. The DNA sequencing result of StAPI5 gene in the expression vector pRI201-AN using vector-specific primer M13 forward by Macrogen company using the Sanger technique

## Slide 2
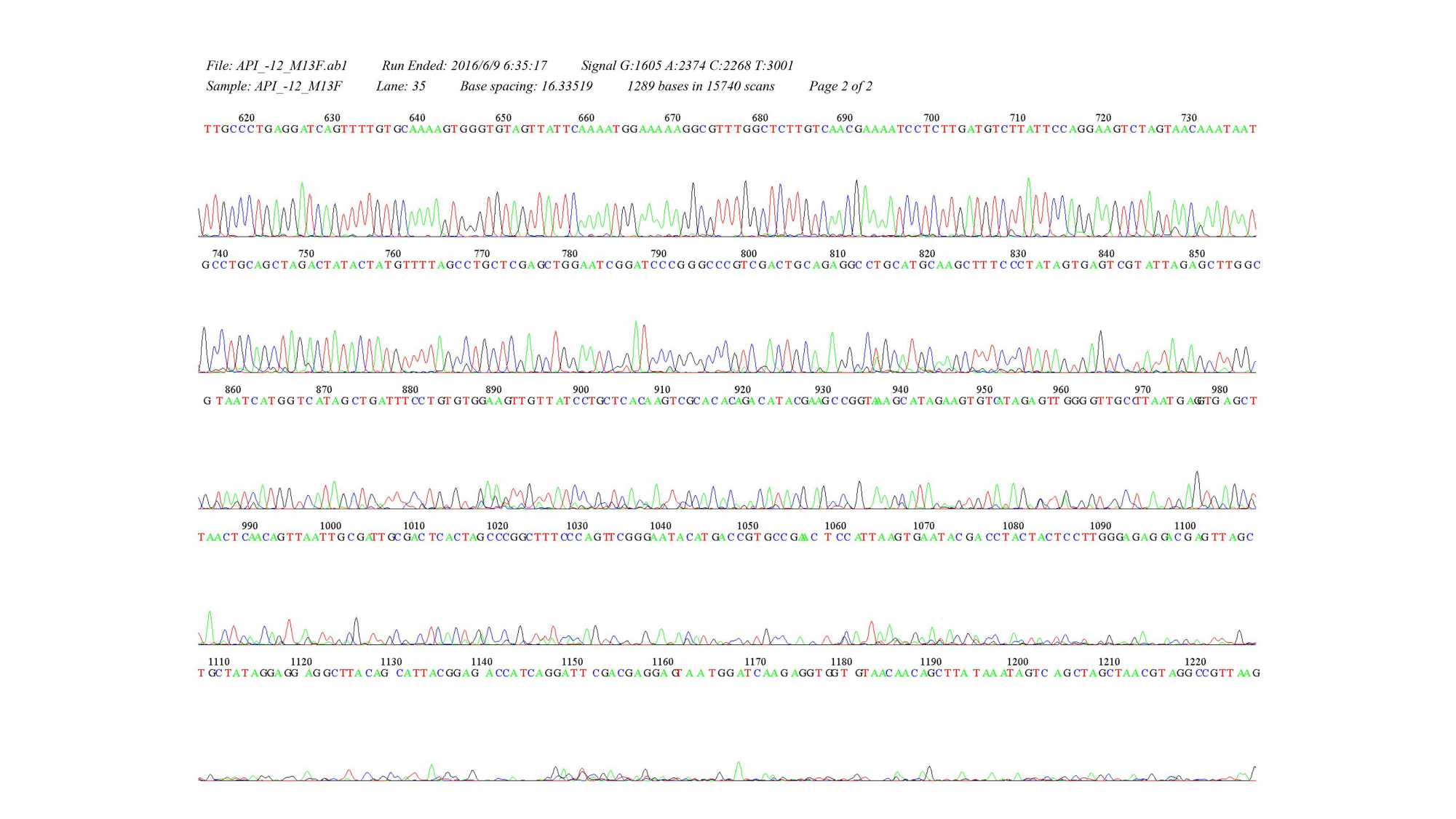

Supplement: Supplementary file 1 — Additional file 1: Figure S1. The DNA sequencing result of StAPI5 gene in the expression vector pRI201-AN using vector-specific primer M13 forward by Macrogen company using the Sanger technique. [file 12870_2022_3596_MOESM1_ESM.pptx]
