# Supplementary material for: Aspartic protease inhibitor enhances resistance to potato virus Y and A in transgenic potato plants
Source: BMC Plant Biol. 2022 May 12;22:241. doi: 10.1186/s12870-022-03596-8 (PMC9097181; doi:10.1186/s12870-022-03596-8)
Supplement: Supplementary file 2 — Additional file 2: Fig. S2. PCR analysis of DNA isolated from transgenic plants with CaMV 35 S forward primer (35S-F) and StAPI5 gene reverse specific primer (StAPI5-R) to verify the insertion of the target gene into the potato genome. The expected band length is 754-bp. C: Negative control with non-transgenic potato plant (wild-type); P: positive control PCR reaction with plasmid as template; Lanes no. 1–10: DNA from independent transgenic potato plants and M: 1 kb DNA Ladder. [file 12870_2022_3596_MOESM2_ESM.docx]

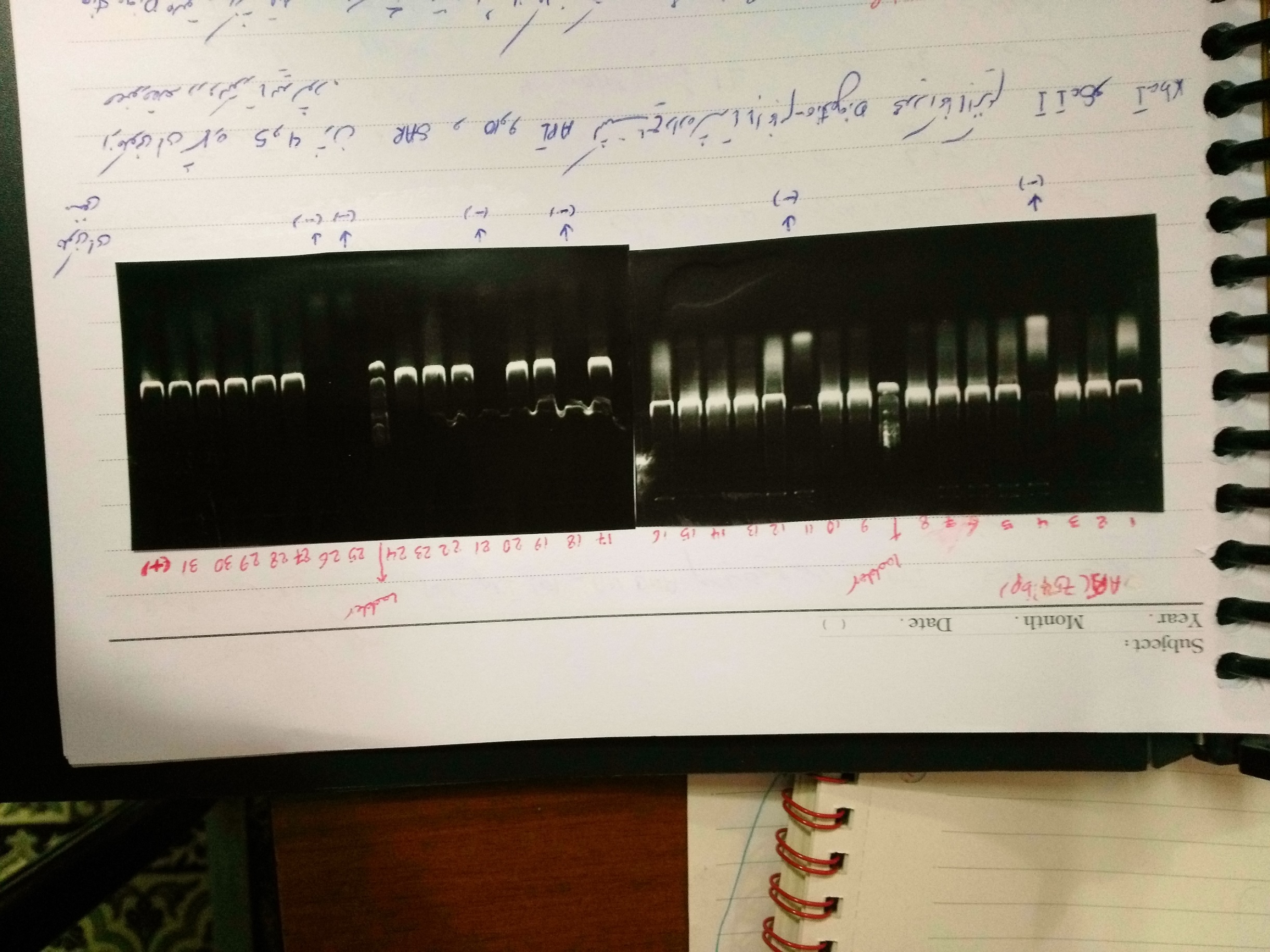

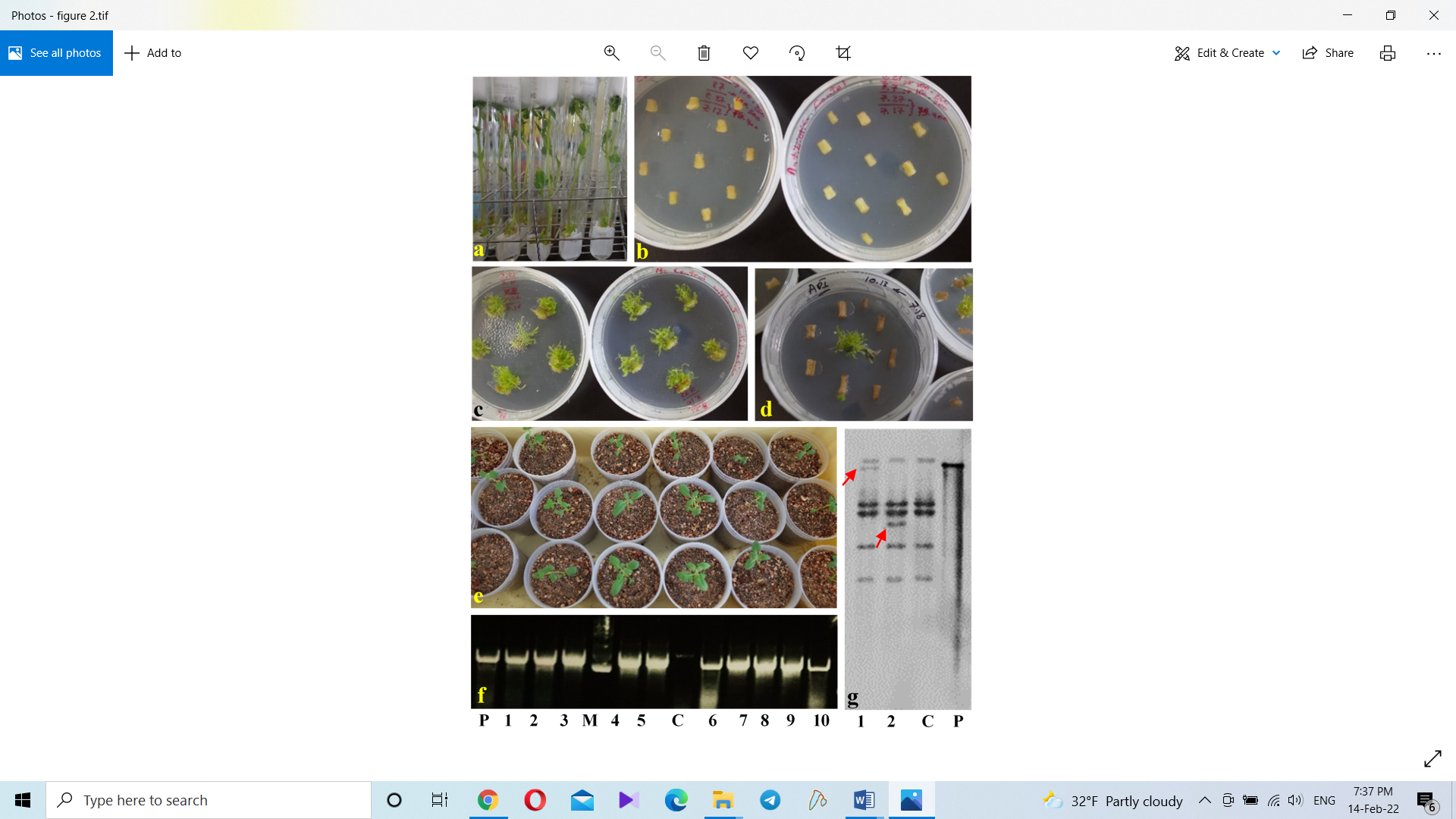


Fig. S2) PCR analysis of DNA isolated from transgenic plants with CaMV 35 S forward primer (35S-F) and *StAPI5* gene reverse specific primer (*StAPI5*-R) to verify the insertion of the target gene into the potato genome. The expected band length is 754-bp. C: Negative control with non-transgenic potato plant (wild-type); P: positive control PCR reaction with plasmid as template; Lanes no. 1–10: DNA from independent transgenic potato plants and M: 1 kb DNA Ladder.
