# Supplementary material for: Aspartic protease inhibitor enhances resistance to potato virus Y and A in transgenic potato plants
Source: BMC Plant Biol. 2022 May 12;22:241. doi: 10.1186/s12870-022-03596-8 (PMC9097181; doi:10.1186/s12870-022-03596-8)
Supplement: Supplementary file 3 — Additional file 3: Fig. S3. Southern blot analysis of the independent transgenic potato plants overexpressing StAPI5 gene (right) in this image and one other studied gene (left) to confirm the transgene integration into the potato genome and to estimate the transgene copy number. P: the pRI-AN201 binary vector containing the StAPI5 gene (positive control); lanes 1 and 2: independent transgenic potato plants; C: non-transformed potato (negative control) [file 12870_2022_3596_MOESM3_ESM.docx]

Fig. S3) Southern blot analysis of the independent transgenic potato plants overexpressing *StAPI5* gene (right) in this image and one other studied gene (left) to confirm the transgene integration into the potato genome and to estimate the transgene copy number. P: the pRI-AN201 binary vector containing the *StAPI5* gene (positive control); lanes 1 and 2: independent transgenic potato plants; C: non-transformed potato (negative control)


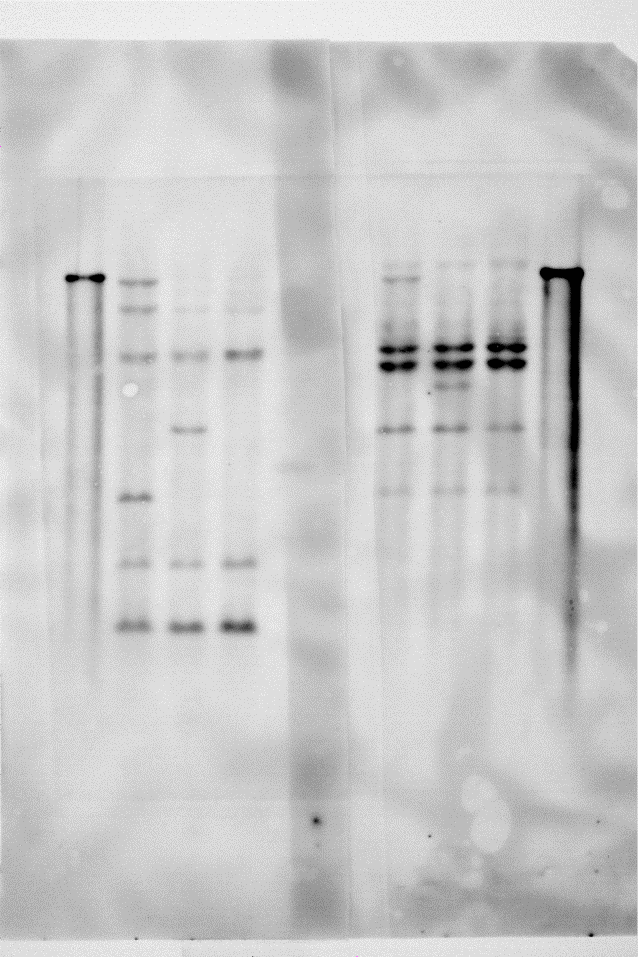

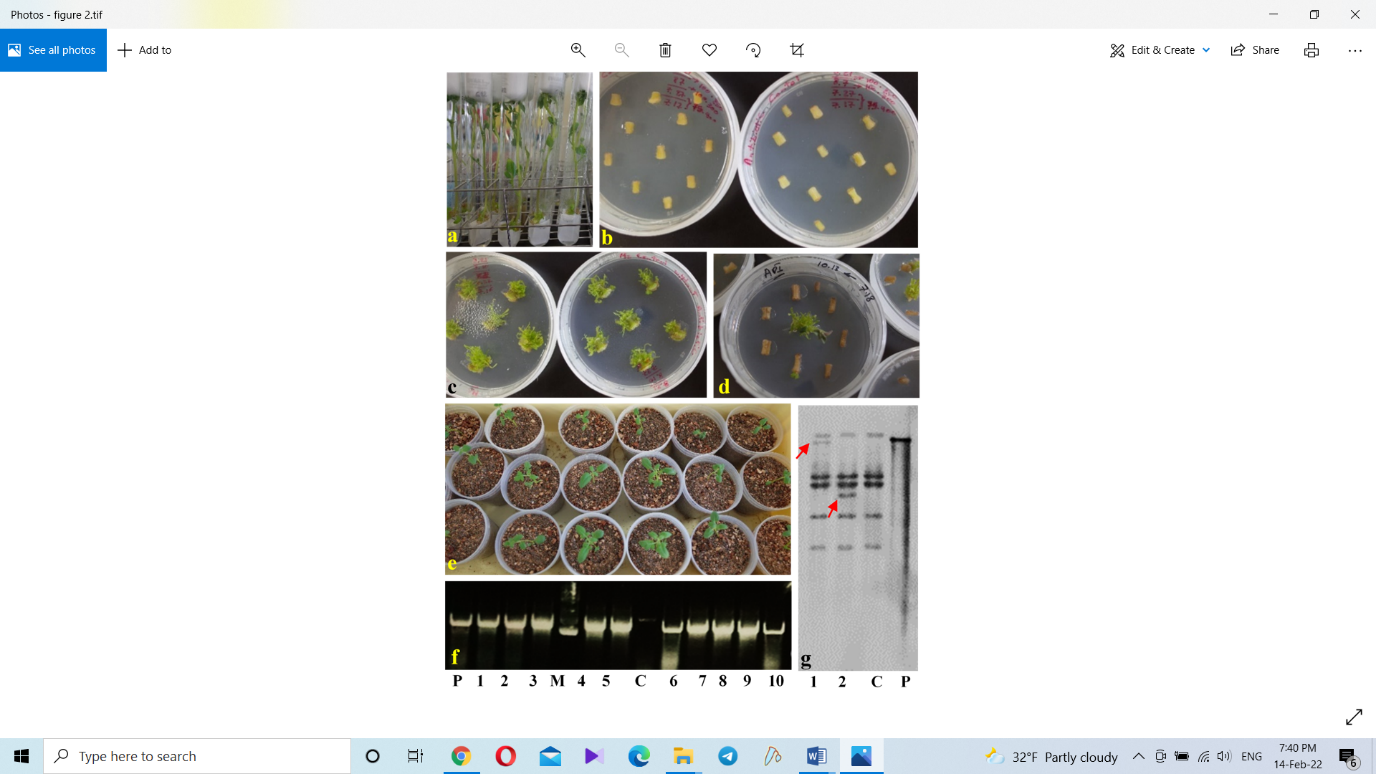


.
