# Supplementary material for: Aspartic protease inhibitor enhances resistance to potato virus Y and A in transgenic potato plants
Source: BMC Plant Biol. 2022 May 12;22:241. doi: 10.1186/s12870-022-03596-8 (PMC9097181; doi:10.1186/s12870-022-03596-8)
Supplement: Supplementary file 4 — Additional file 4: Fig. S4. Pearson’s correlation coefficient between the expression level of StAPI5 and the viral CP accumulation level (significant at P < 0.01). [file 12870_2022_3596_MOESM4_ESM.docx]

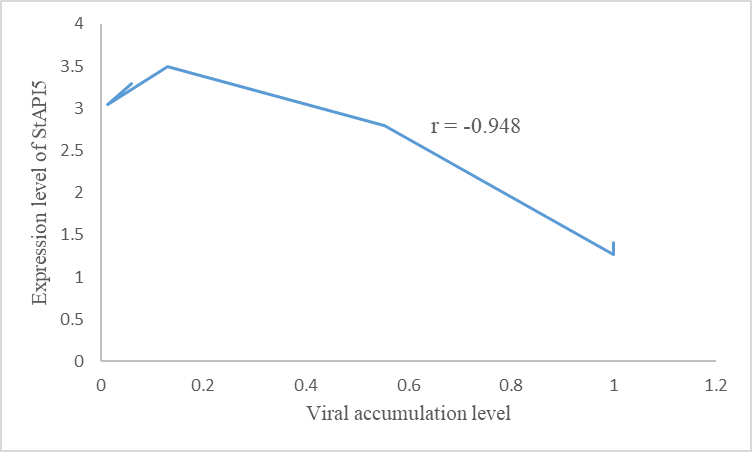


Fig. S4. Pearson’s correlation coefficient between the expression level of *StAPI5* and the viral CP accumulation level (significant at *P* < 0.01).
