# Supplementary material for: Aspartic protease inhibitor enhances resistance to potato virus Y and A in transgenic potato plants
Source: BMC Plant Biol. 2022 May 12;22:241. doi: 10.1186/s12870-022-03596-8 (PMC9097181; doi:10.1186/s12870-022-03596-8)
Supplement: Supplementary file 5 — Additional file 5: Fig. S5. Comparison of the growth of transgenic (StAPI5-OE) and non-transgenic potato plants (Wild-type) at the same developmental stages (8-week-old plants). [file 12870_2022_3596_MOESM5_ESM.docx]

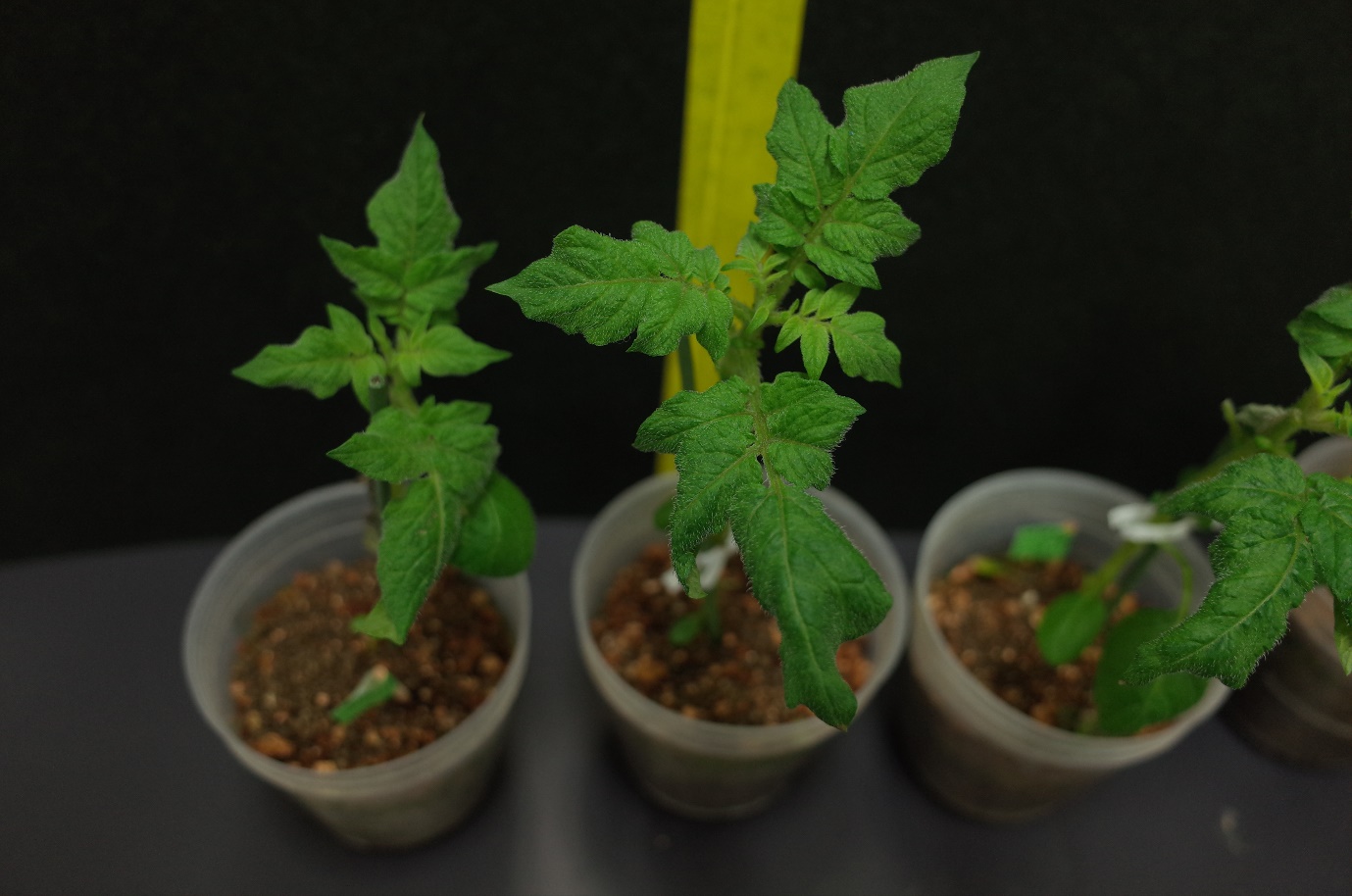


Fig. S5. Comparison of the growth of transgenic (*StAPI5-*OE) and non-transgenic potato plants (Wild-type) at the same developmental stages (8-week-old plants)

***StAPI5*-OE**

**Wild-type**
