# Supplementary material for: Aspartic protease inhibitor enhances resistance to potato virus Y and A in transgenic potato plants
Source: BMC Plant Biol. 2022 May 12;22:241. doi: 10.1186/s12870-022-03596-8 (PMC9097181; doi:10.1186/s12870-022-03596-8)
Supplement: Supplementary file 6 — Additional file 6: Table S1. Values of gas exchange, chlorophyll fluorescence parameters and morphological traits in the resistant cv. Degima, transgenic, and Wilde-type cv. Desiree inoculated with PVA (R/PVA, StAPI5-OE/PVA, and WT/PVA, respectively), PVY (R/PVY, StAPI5-OE/PVY, and WT/PVY), buffer (R/Mock, StAPI5-OE/Mock, and WT/Mock), and without inoculation (R, StAPI5-OE, and WT). The number of replicates in each plant for each treatment was shown as Rep. No. The variables were measured in 21-day-old plants in the growth chamber. Values represent the mean (± SE) of the replicates per treatment. Values within the same row followed by the same letter (s) are not significantly different (Duncan’s multiple range test, P < 0.05). [file 12870_2022_3596_MOESM6_ESM.docx]

| Table S1. Values of gas exchange, chlorophyll fluorescence parameters and morphological traits in the resistant cv. Degima, transgenic, and Wilde-type cv. Desiree inoculated with PVA (R/PVA, *StAPI5*-OE/PVA, and WT/PVA, respectively), PVY (R/PVY, *StAPI5*-OE/PVY, and WT/PVY), buffer (R/Mock, *StAPI5*-OE/Mock, and WT/Mock), and without inoculation (R, *StAPI5*-OE, and WT). The number of replicates in each plant for each treatment was shown as Rep. No. The variables were measured in 21-day-old plants in the growth chamber. Values represent the mean (± SE) of the replicates per treatment. Values within the same row followed by the same letter (s) are not significantly different (Duncan's multiple range test, *P* < 0.05) | | | | | | | | | | | | |
| --- | --- | --- | --- | --- | --- | --- | --- | --- | --- | --- | --- | --- |
| R/PVA | R/PVY | R/Mock | R | *StAPI5*-OE/PVA | *StAPI5*-OE/PVY | *StAPI5*-OE/Mock | *StAPI5*-OE | WT/PVA | WT/PVY | WT/Mock | WT | **Genotype** |
| 3 | 3 | 6 | 3 | ***** | ***** | 3 | 3 | 3 | 3 | 6 | 3 | **Rep. No.** |
| 72.7 ± 1.45  b | 79.0 ± 0.58 a | 74.0 ± 0.63 b | 74.0 ± 1.15 b | 56.7 ± 1.19 e | 61.2 ± 0.94 d | 67.3 ± 0.88 c | 67.3 ± 0.88 c | 11.0 ± 1.15 f | 9.0 ± 0.58 f | 67.0 ± 0.37 c | 67.3 ± 0.67 c | Number of node |
| 140.3 ± 0.88 a | 144.3 ± 0.33 a | 143.2 ± 0.79 a | 142.3 ± 1.45 a | 129.2 ± 1.28 c | 129.9 ± 0.67 c | 134.3 ± 0.33 b | 134.7± 0.67 b | 82.7 ± 1.45 d | 75.7 ± 1.2 e | 133.8 ± 0.40 b | 134.0 ± 0.58 b | Stem length (cm) |
| 1.77 ± 0.09 e | 1.80 ± 0.06 e | 1.42 ± 0.07 f | 1.43 ± 0.09 ef | 2.58 ± 0.05 c | 2.20 ± 0.09 d | 2.33 ± 0.12 cd | 2.33 ± 0.16 cd | 3.53 ± 0.15 b | 3.97 ± 0.09 a | 2.35 ± 0.08 cd | 2.40 ± 0.15 cd | Internode length (cm) |
| 10.0 ± 0.58 b | 11.0 ± 0.58 ab | 11.1 ± 0.37 a | 11.3 ± 0.88 a | 8.3 ± 0.12 c | 7.9 ± 0.09 c | 8.6 ± 0.32 c | 8.6 ± 0.35 c | 3.9 ± 0.06 d | 3.7 ± 0.12 d | 8.4 ± 0.13 c | 8.6 ± 0.26 c | Stem diameter (mm) |
| 147.2 ± 0.72 b | 151.4 ± 2.36 a | 148.8 ± 0.57 ab | 147.9 ± 0.09 b | 124.7 ± 0.63 e | 128.8 ± 0.57 d | 136.7 ± 0.88 c | 136.8 ± 1.01 c | 48.5 ± 1.44 g | 58.1 ± 1.34 f | 136.5 ± 0.55 c | 136.5 ± 0.97 c | Fresh weight (g) |
| 14.6 ± 0.18 a | 15.4 ± 0.41 a | 14.9 ± 0.10 a | 14.7 ± 0.22 a | 11.5 ± 0.16 c | 11.2 ± 0.17 c | 12.8 ± 0.28 b | 12.8 ± 0.25 b | 5.9 ± 0.12 e | 7.7 ± 0.39 d | 12.4 ± 0.16 b | 12.6 ± 0.32 b | Dry weight (g) |
| 4.07 ± 0.09 ab | 4.33 ± 0.09 a | 4.27 ± 0.06 a | 4.23 ± 0.15 ab | 2.80 ± 0.13 c | 2.89 ± 0.18 c | 3.67 ± 0.07 b | 3.73 ± 0.03 ab | 1.73 ± 0.09 d | 1.57 ± 0.18 d | 3.73 ± 0.03 b | 3.77 ± 0.03 ab | leaf area index (LAI) |
| 0.19 ± 0.01 de | 0.19 ± 0.01 de | 0.18 ± 0.004 e | 0.17 ± 0.01 e | 0.34 ± 0.02 b | 0.30 ± 0.01 c | 0.23 ± 0.02 de | 0.23 ± 0.02 de | 0.65 ± 0.01 a | 0.64 ± 0.03 a | 0.26 ± 0.01 d | 0.26 ± 0.01 cd | F´v/F´m |
| 0.90 ± 0.01 b | 0.90 ± 0.02 b | 0.93 ± 0.01 a | 0.937 ± 0.01 a | 0.896±0.003 b | 0.893 ± 0.002 bc | 0.883±0.003 bcd | 0.89 ±0.003 bcd | 0.840 ± 0.01 e | 0.83 ± 0.01 e | 0.878 ± 0.003 d | 0.88 ± 0.01 cd | Fv/Fm |
| 0.65 ± 0.02 cd | 0.67 ± 0.01 bc | 0.61 ± 0.01 d | 0.60 ± 0.01 d | 0.70 ± 0.01 b | 0.70 ± 0.01 b | 0.71 ± 0.02 b | 0.71 ± 0.02 b | 0.80 ± 0.01 a | 0.81 ±0.003 a | 0.71 ± 0.01 b | 0.71 ± 0.02 b | qN |
| 0.22 ± 0.01 ab | 0.24 ±0.003 a | 0.23 ±0.004 a | 0.24 ±0.003 a | 0.16 ± 0.01 d | 0.18 ± 0.01 c | 0.20 ± 0.01 abc | 0.20 ±0.003 abc | 0.07 ± 0.01 e | 0.04 ± 0.02 e | 0.19 ± 0.003 bc | 0.19 ± 0.01 bc | Stomatal conductivity  (mol m^-2^ s^-1^) |
| 2.34 ± 0.09 a | 2.42 ± 0.14 a | 2.28 ± 0.02 a | 2.26 ± 0.01 a | 0.91 ± 0.21 b | 0.47 ± 0.11 b | 1.93 ± 0.1 a | 1.90 ± 0.08 a | 0.44 ± 0.06 b | 0.41 ± 0.05 b | 1.90 ± 0.06 a | 1.90 ± 0.08 a | Transpiration  (mol m^-2^ s^-1)^ |
| 8.25 ± 0.21 a | 8.32 ± 0.5 a | 8.18 ± 0.18 a | 8.17 ± 0.22 a | 6.91 ± 0.3 b | 7.35 ± 0.18 ab | 7.30 ± 0.19 ab | 7.30 ± 0.19 ab | 3.82 ± 1.16 c | 3.43 ± 0.47 c | 7.26 ± 0.3 ab | 7.29 ± 0.19 ab | Net photosynthetic |
| 231.5 ± 1.08 a | 222.0 ± 1.15 b | 231.3 ± 0.69 a | 233.9 ± 0.43 a | 214.8 ± 0.49 d | 216.7 ± 0.60 c | 220.9 ± 0.57 b | 220.9 ± 0.59 b | 202.1 ± 1.55 e | 203.9 ± 2.65 e | 220.6 ± 0.51 b | 220.7 ± 0.72 b | Intercellular CO2 concentration (µmol mol^-1^) |
| 24.4 ± 0.12 cd | 24.11 ± 0.04 de | 24.84 ± 0.01 c | 24.86 ± 0.02 c | 23.97 ±0.1 e | 23.58 ± 0.13 f | 24.05 ± 0.05 de | 24.09 ± 0.06 de | 26.13 ± 0.1 b | 26.79 ± 0.19 a | 24.11 ± 0.04 de | 24.08 ± 0.07 de | The leaf temperature |
| * Five events with 6-9 replicates | | | | | | | | | | | | |
