# Supplementary material for: Aspartic protease inhibitor enhances resistance to potato virus Y and A in transgenic potato plants
Source: BMC Plant Biol. 2022 May 12;22:241. doi: 10.1186/s12870-022-03596-8 (PMC9097181; doi:10.1186/s12870-022-03596-8)
Supplement: Supplementary file 7 — Additional file 7: Table S2. Sequences of the gene-specific, coat protein of PVY and PVA, and internal control primers. [file 12870_2022_3596_MOESM7_ESM.docx]

| Table S2. Sequences of the gene-specific, coat protein of PVY and PVA, and internal control primers | | |
| --- | --- | --- |
| Reverse (5′-3′) | Forward (5′-3′) | Gene |
| CAGCAGGCAGGCTAAAACATAGTAT | GTCTCGAGAAAACACAAGCAATAAAATC | *API5* |
| AGGGCAACGTAGACAAATAATAGAAGTA | TGGAAAGTCGGAAATCTAAATGCATATT | *API5*^*^ |
| AATTCTAATACGACTCACTATAGGG CCAGCTCGAGCAGGCTAAAACATAG | GTCTCGAGAAAACACAAGCAATAAAATC | *API5*^**^ |
| CCATCCATCATAACCCAAACTC | ATACTCGGGCAACTCAATCACA | PVY^*^ |
| TGACATTTCCGTCCAGTCCAA | TTTCTATGAGATCACTGCAACCACT | PVA^*^ |
| CGGTTCTTGATTAATGAAAACATCCT | GGGGCATTCGTATTTCATAGTCAGAG | *18S*^*^ |
| ^*^ primer-sequences used for real-time PCR and ^**^ Southern-blot analysis with T7 promoter at the 5′-end of the gene-specific reverse primer, whereby promoter-sequence is underlined. | | |
